# Action mechanism and molecular design of indolepyrrodione inhibitors targeting IDO1

**DOI:** 10.3389/fmolb.2025.1661700

**Published:** 2025-10-23

**Authors:** Xinmin Wang, Zhigang Zhang, Kaixuan Hu, Wentong Yu, Yan Cheng, Yuting Song, Xin Sun, Siyao Li, Tiantian Yang, Jianping Hu, Jing Jing, Ting Luo

**Affiliations:** ^1^ Laboratory of Integrative Medicine, Clinical Research Center for Breast, State Key Laboratory of Biotherapy, West China Hospital, Sichuan University and Collaborative Innovation Center, Chengdu, Sichuan, China; ^2^ Key Laboratory of Medicinal and Edible Plants Resources Development of Sichuan Education Department, School of Pharmacy, Chengdu University, Chengdu, China

**Keywords:** IDO1, molecular dynamic simulation, conformation change, inhibition mechanism, drug design

## Abstract

**Introduction:**

The incidence of cancer remains high, representing not only a major health threat to humanity but also a substantial economic burden to society. While conventional therapies include surgery, radiotherapy, and chemotherapy, immunotherapy—particularly immune checkpoint inhibitors (ICIs)—has emerged as a promising approach to enhance anti-tumor immunity. Indoleamine 2,3-dioxygenase 1 (IDO1), a cytoplasmic enzyme that regulates tryptophan catabolism, has become an important target for immunotherapeutic drug development.

**Methods:**

In this study, a series of molecular simulation techniques were employed to investigate the molecular recognition and inhibition mechanisms between representative indolepyrrodione (IPD) inhibitor—specifically PF-06840003—and IDO1. Molecular dynamics simulations and structural analyses were conducted to characterize conformational changes of the IDO1 system. In addition, a 3D-QSAR study was performed on 26 IPD analogs using CoMFA and CoMSIA approaches to establish predictive structure–activity models.

**Results:**

Simulation results revealed that the substrate/inhibitor access channel valve (JK-loop) in the IDO1_apo system adopts an open conformation, which transitions to a closed state upon binding of PF-06840003. The inhibitor forms multiple hydrogen bonds with residues in the active site, restricting JK-loop movement and consequently blocking the substrate L-Trp channel. This also narrows the O_2_/H_2_O molecular passage, reducing the efficiency of molecular entry and exit, and thereby attenuating the enzyme’s catalytic activity. The CoMFA and CoMSIA models exhibited high stability and strong predictive capability, providing reliable insights for further inhibitor optimization.

**Discussion:**

These findings suggest a potential inhibitory mechanism for PF-06840003 and offer valuable structural insights for the rational design of potent IDO1 inhibitors. It should be noted that the inhibitory activity of the designed lead compounds is based solely on computational predictions; experimental validation through *in vitro* and *in vivo* studies is still required to confirm their actual inhibitory effects and pharmacokinetic properties.

## 1 Introduction

In 2020, more than 19 million new cases of malignant tumors were reported worldwide (Allemani et al., 2015; Ferlay et al., 2021; Morgan et al., 2023; Sung et al., 2021), positioning cancer as the second leading cause of death after cardiovascular disease and accounting for approximately 9.9 million fatalities globally (Morgan et al., 2023). Cancer has been treated historically through surgery, radiotherapy, and chemotherapy, each with limitations such as metastasis control and systemic toxicity (Liu et al., 2024). Therefore, Immunotherapy has become a promising approach to treating cancer by stimulating the body’s own immune system. Compared with traditional cancer treatments, immunotherapy offers several advantages, including strong antitumor effects, precise targeting, lower toxicity, and the possibility of personalized treatment (Chen and Mellman, 2017). Despite the remarkable progress of immunotherapy, its effectiveness is frequently constrained by the capacity of tumor cells to evade immune recognition and destruction (Kantoff et al., 2010; Majzner and Mackall, 2018),which refers to the diverse strategies employed by tumor cells to escape immune surveillance and resist immune-mediated elimination. To counteract these processes, intracellular mediators of tumor immune evasion have become promising therapeutic targets for small-molecule drug development. Key examples include immune checkpoint regulators such as PD-1 and PD-L1, as well as enzymatic and signaling proteins like IDO1, RORγt, and specific protein kinases (Lee et al., 2018).

**FIGURE 1:**
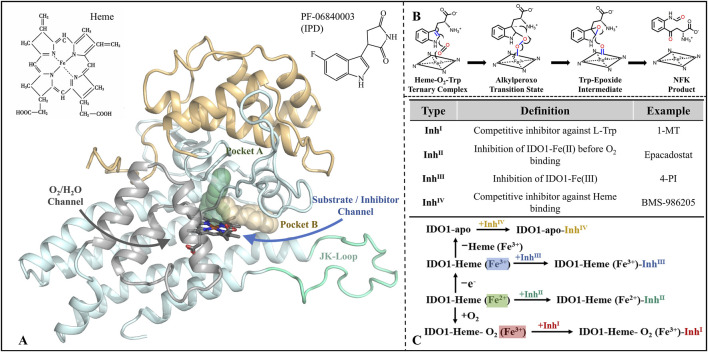
Structure-function of IDO1 and types of inhibitors along with mechanisms of action. **(A)** The three-dimensional architecture of IDO1 comprises two distinct domains: a light blue domain representing the larger structural component, and a yellow domain representing the smaller structural component. The heme molecule is depicted using a stick model, while the cyan Loop region specifically refers to the JK-Loop. The gray section consists of α-helices that form channels for O_2_/H_2_O entry or exit. Positioned above the heme in the active site are Pocket A (depicted in green) and Pocket B (depicted in yellow). **(B)** Conversion of L-Trp catalyzed by IDO1; **(C)** Classification and potential inhibition mechanisms of inhibitors targeting IDO1.

Among these, IDO1 is a heme-containing oxidoreductase that facilitates the enzymatic degradation of the essential amino acid L-Trp to N-formylkynurenine (NFK) *via* the kynurenine pathway (KP) (Sugimoto et al., 2006; Yamazaki et al., 1985), which consists of a small N-terminal domain and a larger C-terminal domain (Röhrig et al., 2021). The C-terminal domain encompasses the enzyme’s catalytic site and structural elements, such as the JK-Loop, which regulate the ingress and egress of substrates and products (Singh and Salunke, 2021). Notably, the flexibility of the JK-Loop is crucial, as it enables the enzyme to adopt various conformations in response to ligand binding, thereby modulating the accessibility of the active site and enzyme activity (Akemi et al., 1991; Fallarino et al., 2006). This conformational flexibility allows IDO1 to effectively control the entry of substrates and the release of products, thereby influencing both its catalytic reactions and regulatory functions (Figure 1). This enzymatic activity suppresses immune responses through three principal mechanisms. First, IDO1-mediated degradation of L-tryptophan (L-Trp) depletes a critical nutrient required for T cell proliferation and differentiation (Munn et al., 2005), with previous studies demonstrating that T cell activation and expansion are markedly impaired when extracellular L-Trp concentrations fall below 0.5–1.0 mM (Godin-Ethier et al., 2011; Munn et al., 2005). Second, intermediate metabolites generated during L-Trp catabolism, including N-formylkynurenine (NFK) and other downstream products, can inhibit T cell function and induce apoptosis (Opitz et al., 2007). Third, IDO1 promotes the differentiation of regulatory T cells (Tregs), which suppress effector T cell proliferation through the secretion of inhibitory cytokines or direct interactions with antigen-presenting cells (Fallarino et al., 2006; Platten et al., 2012). Collectively, these processes enable IDO1 to maintain immune tolerance within the tumor microenvironment, establishing it as a critical target for cancer immunotherapy and other immune-related diseases.

Since the identification of IDO1 as a potential drug target and a key mediator of tumor immune escape, efforts to develop small-molecule inhibitors have intensified (Munn et al., 1998), with inhibitors now commonly classified into four types by their catalytic-site interaction (Röhrig et al., 2019) (see Figure 1C). Type I, exemplified by 1-methyl-L-tryptophan (1-MT), weakly competes with L-Trp in the distal heme (A) pocket and typically does not form a direct Fe coordination bond (Nakashima et al., 2008; Shimizu et al., 1978). Type II, represented by Epacadostat, binds ferrous IDO1 prior to O_2_ entry, coordinating the heme iron *via* its hydroxyamidine oxygen and thereby blocking the subsequent dioxygenation step (Yue et al., 2017). Type III, typified by 4-phenylimidazole (4-PI), directly coordinates the heme iron and inhibits catalysis in the vicinity of the active center (Mautino et al., 2013; Nayak-Kapoor et al., 2018; Sugimoto et al., 2006). Type IV, represented by BMS-986205, exploits reversible heme dissociation to target apo-IDO1, with binding driven predominantly by π–π and hydrophobic interactions, yielding sustained inhibition (Lewis-Ballester et al., 2017). Collectively, these modalities implement occupancy-based control at the catalytic center—either by competing with substrate or oxygen, or by engaging (and, in some cases, promoting dissociation of) the heme cofactor. However, this paradigm is constrained by substrate/O_2_ dependence, heme-coordination off-target liabilities, sensitivity to conformational plasticity and mutations, and uncertain in vitro–in vivo translation (Lewis-Ballester et al., 2017).

Distinct from the four classical, coordination/competition paradigms that rely on “catalytic-center occupancy,” indolepyrrolidinones (IPDs) constitute a non-coordinating class of IDO1 inhibitors. The prototypical IPD, PF-06840003, adopts a crystallographically validated binding pose in which the indole ring nests within pocket A while the succinimide ring lies parallel to the heme plane; notably, it does not coordinate the Fe center, but instead achieves stable, pocket-selective engagement through multiple hydrogen bonds and π–π interactions (Pham et al., 2019). This iron-independent recognition attenuates direct competition with substrate/oxygen and heme-axial sites, potentially improving selectivity and reducing dependence on the enzyme’s redox state and local O_2_ availability (Tang et al., 2021). It simultaneously expands the chemical design space, facilitating rational optimization around non-coordinating pharmacophores (Crosignani et al., 2017). Moreover, PF-06840003 exhibits blood–brain barrier penetration, suggesting therapeutic promise for central nervous system tumors and related indications (Prendergast et al., 2017). Despite structural advances—such as co-crystal elucidation of PF-06840003 bound to IDO1 (Pham et al., 2019)—the dynamic recognition pathway, molecular mechanism of inhibition, and structure-guided design rules for IPD analogues remain insufficiently defined.

We constructed and validated open (IDO1) and closed (IDO1*) conformations of human IDO1 *via* SWISS-MODEL, then ran comparative all-atom MD on four systems (apo IDO1/IDO1* and their PF-06840003 complexes). FELs, time-resolved clustering, and H-bond network analyses reveal ligand-induced JK-Loop inward motion/capping that governs active-site accessibility. HOLE profiling quantifies remodeling of the L-Trp ingress and O_2_/H_2_O channels, supporting a “stable capping → transport limitation” inhibition mechanism. Functionally, this corresponds to restricted substrate entry and reduced catalytic flux, attenuating tryptophan catabolism and its immunosuppressive outputs. To translate mechanism into design, we built CoMFA/CoMSIA 3D-QSAR models for IPD-like chemotypes and, within a free-energy–guided framework, proposed nine synthetically accessible PF-06840003 derivatives. Overall, the study offers testable, structure-informed hypotheses and actionable leads for high-selectivity IDO1 inhibitors. This is a modeling investigation; all mechanistic and activity claims are predictive and require biochemical, cellular, and *in vivo* validation.

## 2 Calculation methods

### 2.1 System preparation

The initial structure employed in this study is based on the IDO1 dimer crystal structure from the PDB database (PDB ID: 5WHR), which encompasses 262 water molecules and protein chains A (E14-E402)/B (S12-K401), along with heme. Both A and B chains exhibit autonomous and complete catalytic functionality. In the 5WHR structure, two JK-Loops lack the P362-E375 region. Initially, the missing portion of the IDO1 structure was reconstructed and validated to ensure its structural rationality, a prerequisite for subsequent MD simulations and even conformational analyses. The target sequence (code: I23O1) and the template (PDB ID: 7M7D/6F0A) were retrieved from the UniProt database (http://www.uniprot.org/) and the Protein Data Bank (http://www.rcsb.org/), respectively. The completion of the missing JK-Loop segment in IDO1 was accomplished using the SWISS-MODEL server. The reconstituted JK-Loop displays two distinct states, namely, open and closed. Furthermore, the JK-Loop exhibits remarkable flexibility by adopting either an open or closed conformation in response to ligands (Röhrig et al., 2021). The constructed model was ultimately evaluated using two methods, namely, Profile-3D (Tomii et al., 2005) and Ramachandran plot (Hooft et al., 1997). In the Profile-3D assessment, a closer proximity of the verified score to the expected high score indicates a higher quality of the modeled structure. Meanwhile, in the Ramachandran plot, a greater distribution of dihedral angles of the modeled structure residues within the most favored region correlates with increased structural reliability.

Specifically, the system retaining protein A chain from 5WHR, heme, and the inhibitor PF-06840003 is denoted as IDO1*_PF, wherein the JK_Loop adopts a closed conformation. By removing PF-06840003 from the IDO1*_PF system, the resulting configuration is referred to as IDO1*_apo. In comparison to IDO1*_PF, the JK_Loop in IDO1_PF-06840003 (IDO1_PF) assumes an open conformation and the removal of the inhibitor yields the IDO1_apo configuration. Evidently, comparing the simulation trajectories of the open-state IDO1_apo and closed-state IDO1*_apo systems reveals the selectivity in the conformation of the JK_Loop in the absence of an inhibitor. By solely analyzing the conformational ensemble of IDO1_PF, insights into whether inhibitor binding induces a transition in the JK_Loop conformation can be gained. Comparing IDO1*_apo with IDO1*_PF provides molecular recognition characteristics of the inhibitor PF-06840003 with the closed-state IDO1 and elucidates its impact on the enzyme’s conformation.

### 2.2 Molecular dynamics simulation

We conducted comparative molecular dynamics simulations of IDO1_apo, IDO1*_apo, IDO1_PF, and IDO1*_PF models for a duration of 300 ns using the AMBER12 software package (Wang et al., 2000) and AMBER ff14SB force field (Wang et al., 2004). The simulation temperature was set to 300 K, and the TIP3P water model (Jorgensen et al., 1983) with a solvent boundary threshold of 10 Å was employed as the solvent model. To maintain electric neutrality in each simulation system, we added 2/2/2/2 Na^+^ ions to their respective models. Additionally, 20942/20160/20943/20147 water molecules were included in each system. The total number of atoms in each system was as follows: 69,109 for IDO1_apo; 66,767 for IDO1*_apo; 69,138 for IDO1_PF; and 66,754 for IDO1*_PF. To ensure the rationality of the initial structure and prevent geometric clashes, two rounds of energy optimization were conducted. Initially, a 5,000 steps steepest descent optimization was performed under the constraint of the solute (with a constraint force constant set to 5.0 × 10^4^ kcal·mol^−1^·nm^−2^), followed by an additional 5,000 steps using the conjugate gradient method. Subsequently, both unconstrained steepest descent and conjugate gradient optimizations were carried out for 5,000 steps each, with a convergence criterion set at an energy gradient less than 4.182 × 10^−4^ kJ·mol^−1^·nm^−2^. The MD simulation consisted of two stages. In the first stage, a 5 ns MD simulation was performed with a solute that was constrained using a constraint force constant set to 1.0 × 10^3^ kcal·mol^−1^·nm^−2^. During this stage, the temperature gradually increased from 0 K to 300 K. The second stage involved an unconstrained isothermal MD simulation lasting for 295 ns. Throughout the MD simulation, bond lengths were constrained using the SHAKE algorithm (Ryckaert et al., 1977), with a non-bonded cutoff set at 1 nm and an integration time step of 2 fs. Additionally, VMD software was utilized to monitor and track the conformational changes of the system during the entire simulation.

### 2.3 Conformational analyses

This study utilizes three methodologies, namely, free energy landscape (FEL), conformational clustering, and residue contact analysis, to dissect the molecular flexibility and dynamic characteristics of both IDO1 and IDO1* as well as investigate the impact of the inhibitor PF-06840003 on the swinging motion of the JK-Loop.

By investigating the spatial distribution of regions exhibiting the lowest folding free energy, FEL offers a comprehensive comprehension of fundamental biological processes such as molecular recognition, folding, and biomacromolecular aggregation under physiological conditions (Nguyen and Derreumaux, 2012). The minimal folding free energy represents an ensemble of conformations that stabilize biomacromolecules in their native states, while free energy barriers indicate transition states connecting these stable conformations. The FEL methodology involves an initial principal component analysis (PCA) of MD simulation trajectories, followed by utilizing PC1 and PC2 as reaction coordinates to construct conformational fluctuation distributions that capture functional slow motions. By leveraging the probabilities derived from these conformational distributions and applying the Boltzmann relation, a predictive formula for estimating free energy is established (Barlow et al., 1993), as shown in Equation 1:

$$∆G\left(X\right)=-k_{B}T\ln P\left(X\right)$$ (1)

In this context, X represents the reaction coordinate, specifically the PC projection values obtained from MD simulation trajectories. $k_{B}$ denotes the Boltzmann constant, T refers to the absolute temperature, and $P\left(X\right)$ signifies the probability distribution of the system’s PCn conformations

Using the MMTSB (Feig et al., 2004) tool, cluster analysis was performed on the MD simulation trajectories of the four systems: IDO1_apo, IDO1*_apo, IDO1_PF, and IDO1*_PF. Following Daura et al.'s approach (Daura et al., 1999), RMSD values between each conformation were systematically calculated to construct an RMSD matrix (N × N, where N is the number of conformations). A predefined RMSD threshold was set, and comparisons were made between RMSD values of any two conformations; if the difference fell below the threshold, they were grouped into a cluster. The representative conformation for each cluster was chosen as the one with the lowest energy. The clustering formula is shown in Equation 2:

$$C=\begin{array}{l} 1\;\left(\text{if}∆\text{RMSD}\leq 0.2\text{ nm}\right)\\ 0\;\left(\text{if}∆\text{RMSD}>0.2\text{ nm}\right) \end{array}$$ (2)

The value of C is set to 1 when the ∆RMSD is below the threshold of 0.2 nm, indicating that two conformations belong to the same cluster; conversely, if C equals 0, it signifies that they belong to different clusters. The remaining unclustered conformations will continue undergoing clustering analysis until all conformations are assigned to specific clusters. The calculation formula for RMSD is shown in Equation 3:

$$\text{RMSD}={\left(\frac{1}{N}\sum_{i=1}^{N}\delta_{i}^{2}\right)}^{1/2}$$ (3)

N represents the total number of atoms, and δi denotes the distance deviation of the *i*th atom in two different conformations.

The residue contact maps were compared based on representative conformations that were clustered together. In this study, residue pairs within a distance of less than 0.45 nm in the IDO1 protein were defined as being in contact with each other. By analyzing the differential contacts between the IDO1_apo, IDO1*_apo, IDO1_PF, and IDO1*_PF systems, we can investigate the impact of inhibitor PF-06840003 binding on protein stability, JK-loop conformation, and local folding. Additionally, this paper defines two parameters - Common contact and Specific contact to characterize the conservation and variation of protein structure upon substrate recognition. The total number of contacting residues (i.e., Common contact + Specific contact) partially reflects protein stability.

### 2.4 Binding free energy calculation

As a crucial parameter in physical chemistry, binding free energy is commonly utilized to evaluate the molecular recognition between receptors and ligands, serving as an essential criterion for assessing the activity of drug molecules (Hogues et al., 2014). In this study, we initially employed the molecular mechanics/Poisson-Boltzmann solvent area (MM/PBSA) algorithm (Kollman et al., 2000) to predict the binding free energies of IDO1 and IDO1* with inhibitor PF-06840003, thereby evaluating the underlying driving force behind their molecular recognition. Subsequently, we utilized Solvated Interaction Energy (SIE) (Naïm et al., 2007) to predict the binding free energies between a series of indolepyrrodione (IPD) inhibitors and IDO1*, aiming to explore molecules with potentially higher inhibitory activity.

In the MM/PBSA method, conformational samples are extracted from stable MD simulation trajectories spanning 180–300 ns. Solvent molecules are subsequently eliminated, and a sample is collected every 5 ns, resulting in a total of 24 solute conformations. The free energy can be partitioned into various energy components, including enthalpy change (∆*H*) and temperature entropy product (*T*∆*S*), wherein the former encompasses vacuum molecular internal energy (∆*E*) and solvent solvation free energy (ΔGsol). The formula for calculating the combined free energy of MM/PBSA is shown in Equation 4:

$$∆G_{\text{cal}}=∆H-T∆S=\left({ELE}_{\text{MM}}+{VDW}_{\text{MM}}+{ELE}_{\text{PB}}+{VDW}_{\text{SA}}\right)-T∆S$$ (4)

Among them, ∆*H* comprises four components: the polar (*ELE*_MM_) and nonpolar (*VDW*_MM_) contributions to the vacuum molecular internal energy, as well as the polar solvation energy (*ELE*_PB_) and nonpolar solvation energy (*VDW*_SA_). T represents the absolute temperature in Kelvin. The entropy change (∆S) characterizes the disorder of receptor folding upon ligand recognition and can be calculated using canonical ensemble methods. When calculating statistical ∆*S*, MD conformations are first optimized for 100,000 steps with an energy gradient convergence criterion of 0.0001 kcal·mol^−1^·Å^−2^.

The SIE method utilizes solvent-accessible surface area to evaluate the affinity and stability between molecules by calculating the energy of molecular interactions. This approach provides an efficient way to screen and prioritize potential drug candidates. Combining the formula for free energy as shown in Equation 5:

$$\text{SIE}\left(\rho ,D_{in},\alpha ,\gamma ,C\right)=\alpha \left[E_{c}\left(D_{in}\right)+\Delta G_{\text{bind}\;}^{R}\left(\rho ,D_{in}\right)+E_{vdw}+\gamma \Delta MSA\left(\rho \right)\right]+C$$ (5)

The binding free energy between a receptor and a ligand, denoted as $\text{SIE}\left(\rho ,D_{in},\alpha ,\gamma ,C\right)$ , is primarily composed of four terms: $E_{c}\left(D_{in}\right)$ describes the Coulombic interactions between solutes; $\Delta G_{\text{bind}\;}^{R}\left(\rho ,D_{in}\right)$ represents the change in reaction field energy calculated using the boundary element program BRIBEM and can be used to represent the polarity component of solvent effects; $E_{vdw}$ accounts for van der Waals interactions between solutes; $\gamma \Delta MSA\left(\rho \right)$ represents the nonpolar component of solvent effects proportional to the change in accessible surface area (Δ*MSA*) upon receptor-ligand binding. Additionally, parameter α serves as a global scaling factor set at 0.1048, representing conformational entropy loss upon binding; parameter $D_{in}$ corresponds to an internal dielectric constant of 2.25 for the solute; parameter ρ is a linear proportionality constant for van der Waals radii with a value of 1.1; parameters γ and C represent the molecular surface area coefficient and correction value, with default values of 1.29 kcal·mol^−1^∙nm^2^ and 2.89 kcal·mol^−1^∙nm^−2^, respectively.

### 2.5 Energy decomposition

The conventional approach for identifying key residues often involves searching for amino acids within a 0.4 nm proximity to the ligand or those involved in hydrogen bonding with the ligand. The former provides information on residues at a distance level, but it is challenging to determine whether these interactions are favorable or unfavorable for binding. The latter relies on stringent geometric criteria, which may overlook some crucial residues. In this study, we utilized molecular mechanics/generalized born surface area (MM/GBSA) energy decomposition (Kollman et al., 2000) method to unveil critical residues involved in receptor-ligand recognition. The fundamental concept of the MM/GBSA method involves partitioning the interaction between each residue in the receptor and ligand into intra-molecular energy (comprising polar electrostatic vacuum and nonpolar van der Waals vacuum) and solvation energy (which encompasses both polar and nonpolar components). These energies are further decomposed for each residue, including their main chains and side chains. Similar to the free energy calculation strategy employed by MM/PBSA, intra-molecular energy is computed using molecular mechanics based on a force field; while polar and nonpolar solvation energies are respectively modeled using the GB model (Tsui and Case, 2000) and LCPO algorithm (Weiser et al., 1999).

### 2.6 HOLE analysis

Channel geometry and accessibility were further analyzed using the HOLE software package to evaluate the impact of inhibitor binding on the O_2_/H_2_O channel in IDO1. HOLE calculates the pore radius along the channel axis by fitting a sphere at each position while avoiding steric clashes with surrounding residues. The analysis provides a profile of the channel radius along its length, allowing the identification of constriction points and regions affected by inhibitor binding. In addition, the axial distribution of residues lining the channel was determined to correlate structural features with functional accessibility.

### 2.7 Transfer entropy analysis

Transfer entropy (TE) was employed to quantify directional information flow between residues. Residue-wise Cα fluctuations were used to calculate normalized transfer entropy (NTE), capturing causal and asymmetric dependencies in signal propagation. Global NTE was computed by summing residue-to-residue contributions, while regional NTE was analyzed by grouping residues into functionally relevant segments. TE calculations were performed using an in-house Python script based on the Gaussian Network Model (GNM), which takes a dynamically stable PDB structure as input.

### 2.8 Three-dimensional quantitative structure-activity relationship (3D-QSAR)

The 26 IPD inhibitor molecules were randomly divided into a training set (consisting of 22 compounds) for the construction of the 3D-QSAR model and a test set (comprising 4 compounds) to evaluate the predictive capability of the model on biological activity. The experimental IC_50_ values were transformed into negative logarithmic pIC_50_ form. Prior to constructing the 3D-QSAR model, alignment was performed on compound molecules. Considering that the 3D-QSAR model assumes all molecules bind to the same active site in a uniform manner, Cmpd #23, which possessed crystallographic experimental data, was selected as the template molecule. The indole ring served as the common scaffold for alignment, and molecular docking was employed to generate conformations for alignment purposes. Molecular docking experiments were performed using AutoDock 4.2 software package, in which the sampling is based on Lamarckian genetic algorithm (LGA), and the scor-ing adopts the semi-empirical function of the binding free energy (Hu et al., 2013). The size of the docking rectangular box is 40 Å × 40 Å × 40 Å with a grid spacing of 0.375 Å, the box central position was set as: X-11.441, Y-30.878 and Z-32.253. The parameter of GALS runs was set as 128 in all docking, and the structure with the lowest energy in the largest cluster was taken as the final complex model. After screened out, we will check the interactions between ligands and substrate and IDO1 to test the rationality of the molecular docking results.

The alignment of all molecules for 3D-QSAR model construction was performed based on the common benzpyrole. An RMSD cutoff of 2.0 Å was applied for the atomic superposition. This threshold was selected to ensure a reasonable geometric overlap of the key hydrophobic core while allowing for minimal conformational flexibility in the substituent groups, which is consistent with the observed SAR data. Molecules that could not be aligned within this RMSD constraint were excluded from the final model to maintain alignment quality. The SYBYL-X 2.0 (Dubey and Kalra, 2013) software was utilized for conducting the analysis. The CoMFA model was established by placing aligned IPD molecules in a spatial grid, where sp3-hybridized C^+^ probe particles explored the surroundings of the molecule. Interactions between the probe particles and the molecules were computed to obtain energy values at various spatial coordinates, which were used to derive the steric field (S) and electrostatic field (E). In addition to the CoMFA calculation procedure, CoMSIA further investigated and calculated the hydrophobic field (H), H-bond acceptor (A), and H-bond donor (D) around the molecules.

The analysis of the training set using Partial Least Squares (PLS) involved a two-step process. Firstly, leave-one-out (LOO) cross-validation was performed with default parameters to determine the optimal number of components (ONC) and coefficient of determination (*q* ^2^). Secondly, a non-cross-validated 3D-QSAR model was established based on the identified ONC to obtain correlation coefficient (*r* ^2^), standard deviation (*E*_s_), root means squared error (RMSE), and significance coefficient (*F*). The obtained PLS parameters were utilized for assessing the predictive capability and stability of the model as well as predicting biological activity in test molecules. The predicted coefficient $r_{p}^{2}$ is calculated using Equation 6:

$$r_{p}^{2}=\frac{\text{SD}-\text{PRESS}}{\text{SD}}$$ (6)

Here, SD represents the sum of squared deviations between the experimental biological activity values of molecules in the test set and the average experimental biological activity value of molecules in the training set. PRESS represents the sum of squared errors between the predicted biological activity values of molecules in the test set and their corresponding experimental biological activity values.

## 3 Results and discussion

### 3.1 Structural biology survey of IDO1

Up to now, the Protein Data Bank contains 70 crystal structures of IDO1-ligand complexes, which were obtained by X-ray method and are all human proteins. Supplementary Table S1 shows the bioinformatics data for the available IDO1 structures, with resolutions ranging from 1.65 to 3.45 Å. Of all 70 structures, the number of IDO1 dimers and monomers was 13 and 57, respectively (18.6% and 81.4%, respectively). The minimal functional unit of IDO1 was demonstrated to be the monomer by Pham et al. (2019). At present, the molecular recognition and inhibition mechanism of IPD inhibitors and IDO1 is still unknown, in order to better reveal the interrelationship between both, and then provide assistance for the design of novel inhibitors, this work chose the crystal structure of the complex containing IDO1 and PF-06840003 (5WHR) as the basis for a series of molecular mechanics studies.

### 3.2 IDO1 structure completion

**FIGURE 2:**
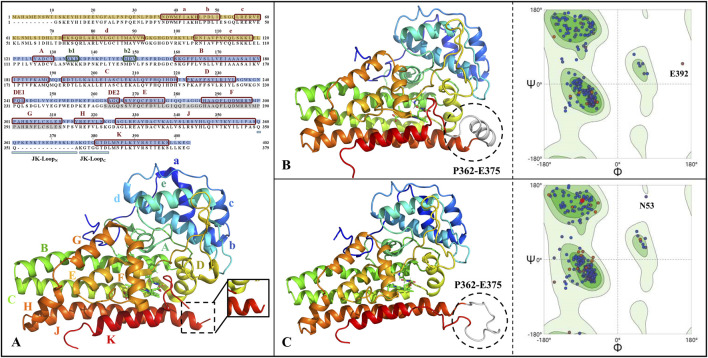
Primary sequence alignment and structural completion and validation of IDO1. **(A)** Sequence alignment and three-dimensional structure, with subdomains of small helical structures labeled as a-e and large helical structures labeled as A-H&JK; The JK-Loop, highlighted in cyan, exhibits structural deficiency from P362 to A375; α-helices and β-sheets are denoted by red and green boxes, respectively; The N-terminal and C-terminal domains are filled in yellow and blue, respectively; Residues shaded in gray represent the O_2_/H_2_O small molecule channel in the closed state of IDO1. The full-length structures of IDO1 in the closed state **(B)** and open state **(C)** along with their Ramachandran distributions, where the completed JK-Loop is depicted in white. Deep green and light green represent the most favored and allowed regions for residue conformations, respectively, while the remaining areas indicate the disallowed region. The majority of residues are distributed in the most favored region, suggesting the reliability of the completed three-dimensional structure of IDO1.

Figure 2 gives the primary sequence as well as tertiary structure complementation and validation of IDO1. It can be seen that the human-derived IDO1 consists of 403 amino acids, in which the JK-Loop part of the structure P362-A375 is missing. Comparing the closed and open state structures of IDO1, the JK-Loop of the former presents a relatively stable α-helix structure and covers the pathway to the active site through a section of hairpin structure, while the JK-Loop of the latter stretches out in an aqueous environment, exposing the active site located above the heme. Based on the 3D structure of IDO1 after JK-Loop structural complementation, model reasonableness was assessed by Ramachandran distribution and Profile-3D scoring. As shown in Figures 2B,C, 96.9%/98.4% of residues in IDO1 and IDO1* models are located in the favored regions, with only 0.3% in disallowed regions, but their positions are distant from the binding pocket and are unlikely to affect ligand interactions. Overall, the complemented structure demonstrates reasonable stereochemistry suitable for further molecular simulations.

*Both IDO1/IDO1* models showed Verify scores (188.6/187.6) exceeding the expected high values (∼178), further supporting model reliability (Supplementary Figure S1). All residues yielded positive Verify scores except for E356 in IDO1*, which was negative but not located near the binding pocket. Together with the Ramachandran results, these findings strongly support the validity of the completed IDO1 and IDO1* models.

### 3.3 Convergence characteristics of MD simulation trajectories

Prolonged molecular dynamics simulations were performed to explore the substrate recognition mechanism of IDO1. As shown in Supplementary Figure S2A, the potential Energy of the four systems converges to equilibrium after 10 ns, indicating the reasonableness of the MD simulations and the reliability of the trajectories. In Supplementary Figure S2B, from the RMSD analysis (Supplementary Figure S2B), IDO1*_apo (closed JK-Loop without inhibitor) exhibited the lowest deviation (0.19 ± 0.02 nm), indicating the highest stability. In contrast, IDO1_PF (open JK-Loop with PF-06840003) showed the largest deviation (0.34 ± 0.03 nm), suggesting weaker binding of the inhibitor in the open state. IDO1_apo and IDO1*_PF displayed intermediate stability with RMSD values of 0.30 ± 0.04 and 0.21 ± 0.02 nm, respectively. These results indicate that JK-Loop closure contributes significantly to system stability and enhances inhibitor binding. In Supplementary Figure S2C, the residue-level RMSF distributions of the four systems are generally similar. The loop near the active site (A260-A265) shows low and overlapping fluctuations across all systems, indicating rigid conservation required for catalytic function. The O2/H2O channel region (S263-S312) also remains stable upon inhibitor binding, in contrast to the structural alterations reported by Liu’s research (Liu et al., 2020). Compared to the complex system IDO1*_PF the JK-Loop (Q360-D383) is much less flexible, suggesting that the binding of PF-06840003 helps to stabilize and constrain the conformation of this Loop. Finally, the high correlation among the four RMSF profiles (R > 0.65 for all, Supplementary Figure S2D) further supports the reliability of the MD trajectories and subsequent conformational analyses.

### 3.4 Conformational analyses

#### 3.4.1 The overall flexibility of IDO1 is higher than IDO1*, with the binding to PF-06840003 amplifying the difference

**FIGURE 3:**
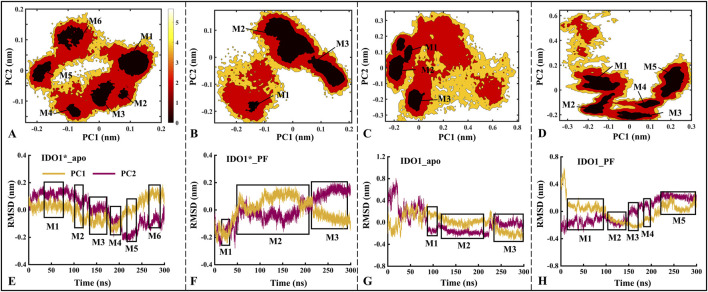
The distributions of the free energy landscape **(A–D)** and the time-dependent changes in the Root Mean Square Deviation (RMSD) of the first (PC1) and second principal components (PC2) are shown for the IDO1*_apo **(A, E)**, IDO1*_PF **(B, F)**, IDO1_apo **(C, G)**, and IDO1_PF **(D, H)**. M1-M6 represent the six major low free energy regions identified in the free energy landscape **(A–D)**. These conformational sub-states correspond directly to the clustered regions in the PCA projection **(E–H)**.The panels **(A–D)** exhibits a higher concentration of conformational numbers as the color darkens, indicating a correspondingly lower conformational free energy. The regions depicted in boxes **(E–H)** represent areas of lower free energy and provide information about simulation time.

Based on MD trajectories, the Free Energy Landscape (FEL) derived from principal component analysis characterizes the conformational space and major functional motions of the systems. Figure 3 illustrates the distribution of the free energy landscape and the variation of RMSD over time for the first (PC1) and second principal components (PC2) for the four systems. The number of low folding free energy regions for IDO1*_apo, IDO1*_PF, IDO1_apo, and IDO1_PF systems are 6, 3, 3, and 5, respectively (see Figures 3A–D). It can be observed that with the binding of the inhibitor PF-06840003, the IDO1* structure becomes more compact, and its motion range decreases; conversely, the open structure of IDO1 may undergo a conformational change.

Further, the time-containing variations of PC1 and PC2 in RMSD are discussed. As shown in Figures 3E–H, the PC1/PC2 rise and fall ranges of IDO1*_apo, IDO1*_PF, IDO1_apo and IDO1_PF are −0.20 to 0.20/ to 0.20 to 0.20, −0.25 to 0.20/−0.25 to 0.20, −0.25 to 0.80/−0.32 to 0.35, −0.35 to 0.30/−0.25 to 0.65 nm, with areas of 0.16, 0.20, 0.74, 0.59 nm^2^. In terms of overall functional motion, IDO1 in the open state rises and falls more than IDO1* (closed state), which is in agreement with the previous RMSD analyses (Supplementary Figure S2B). Interestingly, PC1 exhibited a larger fluctuation range than PC2, suggesting that PC1 captures the dominant functional motion.

#### 3.4.2 IDO1 prefers a closed-state of JK-loop after the association with PF-06840003, otherwise choosing an open-state

**FIGURE 4:**
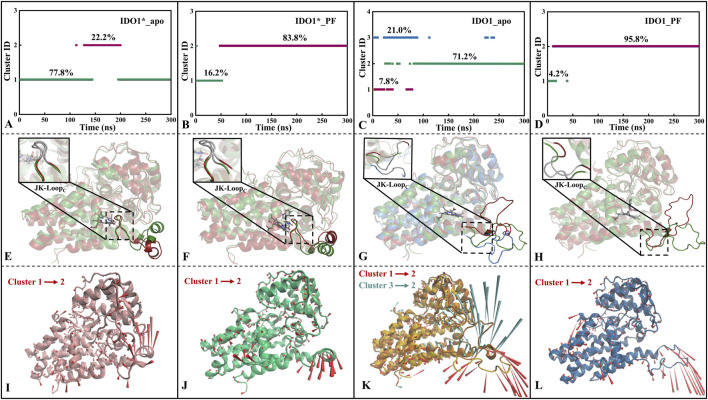
Conformational clustering **(A–D)**, average conformation overlay **(E–H)**, and motion mode analysis **(I–L)** were performed for four systems, namely, IDO1*_apo **(A, E, I)**, IDO1*_PF **(B, F, J)**, IDO1_apo **(C, G, K)**, and IDO1_PF **(D, H, L)**. The cone model was used to describe the motion modes with the arrow direction indicating the movement direction and length representing the magnitude.

After FEL analysis, conformational clustering (Figures 4A–D) was performed for the four systems IDO1*_apo, IDO1*_PF, IDO1_apo and IDO1_PF, respectively, using 0.2 nm as the RMSD cut-off threshold. Except for IDO1_apo, which yielded three clusters (71.2%, 21.0%, 7.8%), all other systems were divided into two clusters (IDO1*_apo: 77.8%, 22.2%; IDO1*_PF: 83.8%, 16.2%; IDO1_PF: 95.8%, 4.2%). The ranking of conformational diversity was therefore IDO1_apo > IDO1*_apo > IDO1*_PF > IDO1_PF, indicating that the open state is more dynamic, while PF-06840003 binding restricts conformational transitions.

As shown Figures 4E,I, JK-Loop_N_ (Q360-A376) of IDO1*_apo exhibits a tendency to open outwards, exposing the substrate entry and exit channels. The cluster jump in system IDO1*_PF is mainly reflected in the JK-Loop_N_ region that further closes the active site channels. In addition, there was no significant change in the conformation of JK-Loop_C_ (K377-D383) in either of the above two systems, which is speculated to be possibly due to the stable hydrogen bond formed between the active site G262 of IDO1*_apo and T379 of JK-Loop_C_ (see Figures 4F,J). According to the conformational and plasticity studies of IDO1_apo by Khoa et al., the GTGG fragment in JK-Loop_C_ is highly conserved and forms a hydrogen bonding network with the active center, which is presumed to be critical for stabilizing the active center substrate (Pham et al., 2019). In both open-state IDO1_apo and IDO1_PF, the former possesses the most frequent tendency to oscillate in and out of the JK-Loop, while the latter exhibits a single inward closure of the substrate channel (see Figures 4G,H,K,L). Comparison of open (IDO1_apo, IDO1_PF) and closed states (IDO1*_apo, IDO1*_PF) suggests that inhibitor binding promotes JK-Loop_C_ stabilization and favors a more ordered, β-hairpin-likeconformation.

#### 3.4.3 PF-06840003 binding contributes to JK-loop closure confirmed by the increase of contact residue number

Based on the representative conformations of each cluster of the four systems, Supplementary Figures S3–S6 show the changes of residue contacts in the representative conformations after the clustering of the four systems. As shown in Supplementary Figure S3, the total number of contacted residues of the system IDO1*_apo decreases from 589 to 567 and is mainly concentrated in JK_Loop, which shows an insufficiently stable conformation. This is consistent with the previous trend of openness towards the closed JK-Loop in IDO1*_apo (see Figure 4I). IDO1*_PF showed the highest conformational conservatism with a common contact residue number of 461, accompanied by a small increase in the total contact number from 591 to 608 (see Supplementary Figure S4). This suggests that the closed-state IDO1*_apo is capable of stabilizing the chelating inhibitor, consistent with the previous low RMSD value (see Supplementary Figure S2B) and smaller folding free energy region number (see Figure 3B). Although the JK-Loop regions of the IDO1_apo system have two oscillatory tendencies, closed and open, and the total number of contact residues remains relatively stable, they are more favorable to JK- Loop region to be outwardly extended and thus fully exposed to the solvent (see Supplementary Figure S5). In Supplementary Figure S6, the total number of contact residues of the IDO1_PF system rises from 565 to 579, indicating that the binding of the inhibitor induces the JK_Loop to exhibit a tendency to be closed.

### 3.5 Molecular recognition of PF-06840003 by IDO1

#### 3.5.1 Non-polar part of enthalpy change is the main recognition driving force

The binding free energy is one of the most importnt physicochemical parameters for evaluating the strength of receptor-ligand interaction and molecular recognition characteristics. In order to compare the difference in recognition ability between the inhibitor PF-06840003 and the closed and open states, the MM/PBSA method was used to predict their binding free energies. As listed Table 1, the enthalpy change (*∆H*) and entropy change (*T*∆*S*) in the IDO1*_PF system were −22.83 and −14.07 kcal·mol^−1^, corresponding to a calculated binding free energy of −8.76 kcal·mol^−1^, which is in good agreement with the experimental value of −9.25 kcal·mol^−1^ (Fallarino et al., 2006). In comparison, the enthalpy change (*∆H*) and entropy change (*T*∆*S*) values between IDO1 and PF-06840003 are −22.9 and −16.28 kcal·mol^−1^, corresponding to a calculated binding free energy of −8.08 kcal·mol^−1^, which is in good agreement with the experimental value of −8.46 kcal·mol^−1^ (Nguyen et al., 2020; Tumang et al., 2016). This indicates that the recognition between IDO1 and PF-06840003 occurs spontaneously and the recognition of the closed-state system is more robust, which is also consistent with the previous FEL, conformational cluster and contact residue analyses. As observed from the Delta column in Table 1, the nonpolar binding energies (*VDW*_MM_ + *VDW*_SA_) of the two systems are −27.35/-27.97 kcal·mol^−1^, respectively, both of which are the main drivers of binding. However, the polarity of the binding energy (*ELE*_MM_ + *ELE*_PB_) as well as the entropy effect (*T*∆*S*) are unfavorable factors. Finally, IDO1_PF exhibits a stronger entropy change than IDO1*_PF, which may be related to the fact that the open-state system has a stronger JK-Loop swing.

**TABLE 1:** The contribution of each energy term in the binding energy of PF-06840003 with IDO1 and IDO1* (kcal·mol^−1^).

Systems	Items	Receptor	PF-06840003	Complex	Delta
IDO1*_PF	*ELE*_MM_	−11901.34 ± 55.74	−99.58 ± 0.98	−12025.81 ± 54.97	−24.89 ± 3.46
	*VDW*_MM_	−1853.60 ± 17.27	0.50 ± 0.36	−1876.78 ± 16.30	−23.68 ± 1.02
	*ELE*_PB_	−3,946.88 ± 51.67	−23.70 ± 0.81	−3,941.15 ± 48.66	29.42 ± 2.23
	*VDW*_SA_	100.34 ± 1.11	3.17 ± 0.01	99.84 ± 1.18	−3.67 ± 0.06
	*∆H*	−7,862.05 ± 132.69	−63.62 ± 2.32	−7,948.50 ± 134.70	−22.83 ± 0.47
	*T*∆*S*	4,466.42 ± 10.52	35.20 ± 0.01	4,487.55 ± 3.60	−14.07 ± 7.91
	∆*G*_*cal*_	−8.76			
	∆*G*_exp_	−9.25			
IDO1_PF	*ELE*_MM_	−11344.34 ± 67.45	−100.65 ± 1.49	−11471.65 ± 68.02	−26.66 ± 4.25
	*VDW*_MM_	−1765.13 ± 29.97	0.76 ± 0.39	−1788.69 ± 29.54	−24.32 ± 2.14
	*ELE*_PB_	−4,115.08 ± 57.02	−23.52 ± 0.30	−4,106.86 ± 57.77	31.74 ± 1.92
	*VDW*_SA_	101.84 ± 0.86	3.16 ± 0.02	101.35 ± 0.84	−3.65 ± 0.04
	*∆H*	−7,715.60 ± 61.77	61.31 ± 3.54	−7,799.81 ± 62.05	−22.90 ± 2.12
	*T*∆*S*	4,356.72 ± 7.19	35.20 ± 0.01	4,375.64 ± 5.86	−16.28 ± 3.71
	∆*G*_*cal*_	−8.08			
	∆*G*_exp_	−8.46			

*ELE*_MM_ and *VDW*_MM_ respectively represent the electrostatic and van der Waals parts of intramolecular energy under vacuum, both of which can be predicted using molecular mechanics. *ELE*_PB_ and *VDW*_SA_ are polar and non-polar parts of solvation-free energy, respectively. The former can be calculated by the PB algorithm, while the latter can be obtained by linear fitting with the solvent-accessible surface area.

#### 3.5.2 The T379-G262 H-bond dominates the inward swing of JK-loop aiding the closure of IDO1 active pocket

**FIGURE 5:**
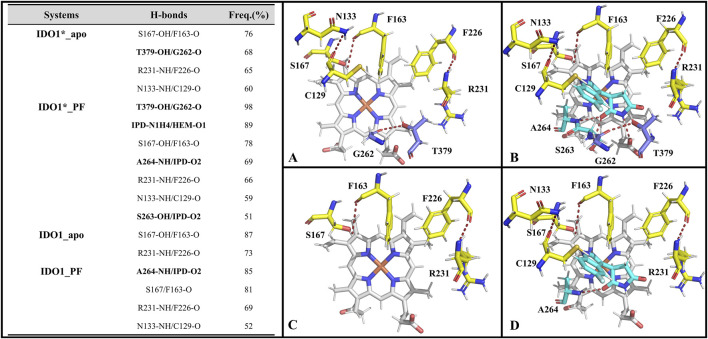
The hydrogen bonds formed by the active pockets of IDO1*_apo **(A)**, IDO1*_PF **(B)**, IDO1_apo **(C)**, and IDO1_PF **(D)** with an occupancy rate exceeding 50%.

We focus on the specific hydrogen-bonding interactions that control JK-loop movement and active-site closure in IDO1, omitting a general overview of hydrogen bonding in secondary-structure stabilization. Hydrogen bonds are defined based on geometric parameters, requiring the distance between the donor and the acceptor to be less than 0.35 nm, and the donor-H-acceptor angle to be greater than 135°. Subsequently, their occupancy rates are calculated based on molecular dynamics trajectories. Figure 5 reports H-bond occupancies >50% across four systems. Overall H-bond counts rank as: IDO1_PF (7) > IDO1_apo (4) ≈ IDO1_PF (4, 1 involving inhibitor) > IDO1_apo (2). Notably, binding of PF-06840003 markedly increases the T379-G262 H-bond occupancy from ∼68% to ∼98% in the closed (IDO1) state (Álvarez et al., 2016) T379 therefore forms a persistent interaction not only with substrate in previous reports but also with G262 on the flexible A260-A265 loop, stabilizing the JK-loop that caps the binding pocket and promoting channel closure. Conversely, in open systems (IDO1_apo and IDO1_PF) the T379-G262 contact is lost and the JK-loop is extended, lowering barrier to ligand entry. We additionally observe PF-induced formation of an N133-C129 H-bond that bends the linker between the large and small domains, bringing domains closer and reducing accessibility to pocket A. Together, these hydrogen-bond rearrangements provide a mechanistic explanation for how PF binding alters local and long-range coupling to lock the JK-loop and inhibit catalytic turnover.

#### 3.5.3 The identified key residues favoring the recognition of PF-06840003 by IDO1 and IDO1*

Supplementary Table S2 lists the key residues in the systems IDO*_PF and IDO_PF that recognize inhibitor binding energy values less than −0.6 kcal·mol^−1^. Overall, there are 8 and 7 key residues in the closed and open state systems, respectively, including 6 identical key residues (i.e., S263, A264, S167, F163, Y126 and L234). Among these key residues, S263 and A264 located on a highly conserved loop (A260-A265) at the active site play significant roles by forming hydrogen bonds with inhibitor PF-06840003 (refer to Figure 5), corroborating with the negative polarity interaction data (*ELE*_MM_ + *ELE*_GB_). Additionally, interestingly, in a functional difference study of S167 on h-IDO1 and TDO (Tryptophan 2,3-Dioxygenase), Chauhan et al. based on kinetic and spectroscopic data found that S167 was able to stabilize the ligand in the presence of a water molecule by providing hydrogen bonding by mutating S167 to alanine and histidine (Chauhan et al., 2008). In addition, the co-crystal structure of PF-06840003 with IDO1 also revealed that the indole-NH of the inhibitor interacts with the side chain of S167 and forms a hydrogen bond; both L234 and V130 form a stable π-alkyl interactions with the benzene ring of the inhibitor, with strong hydrophobic interactions (Alexandre et al., 2018). The biggest difference between the key residues of the two complex systems lies in the T379 of IDO*_PF, which has the ability to form a highly stable hydrogen bond with the JK-Loop region G262 covering the binding site (see Figure 5), having a crucial role in controlling substrate/inhibitor entry and exit channels.

### 3.6 PF-06840003 binding only compresses the O_2_/H_2_O channel without causing a destructive conformational change

Previous studies have shown that PF-0684003 not only weakly recognizes IDO1_apo and induces partial closure of the JK-Loop, but also strongly binds to IDO1*_apo, resulting in a complete closure of the JK-Loop, unfavorable for substrate L-Trp entry. Indeed, in a structural biology study of the IDO1 substrate/inhibitor binding site, Lewis-Ballester et al. reported a narrow channel located between the E-helix and the F-helix that promotes catalytic cleavage of the L-Trp indole ring by mediating the shuttling of oxygen to the catalytic site, closely correlating with the catalytic activity (Lewis-Ballester et al., 2017). In order to investigate whether the binding of PF-06840003 would have an impact on the IDO1 small molecule channel (O_2_/H_2_O) disruption, the HOLE software (OS et al., 1996) package was used to visualize and analyze the active pocket size, including the pocket radius and the axial distribution of the closest amino acids.

**FIGURE 6:**
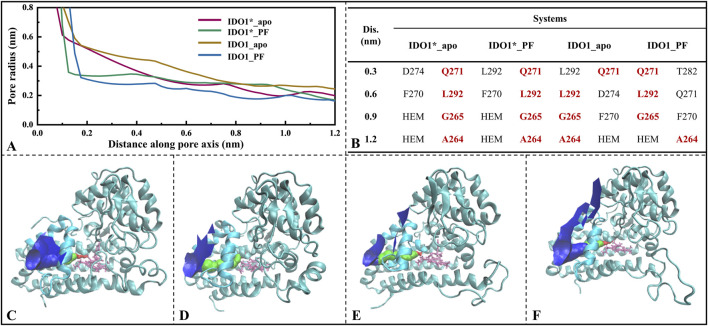
Analysis of O_2_/H_2_O small molecule channels in four systems. **(A)** Variation of channel radius with axial distance, where the position at 1.2 nm on the *x*-axis represents the center of the active pocket; **(B)** Closest residues at different axial distances; O_2_/H_2_O small molecule channels displayed in systems IDO1*_apo **(C)**, IDO1*_PF **(D)**, IDO1_apo **(E)**, and IDO1_PF **(F)**.

Figure 6A gives the variation of the channel axis radius along the channel distance for the four systems. The O_2_/H_2_O small molecule channels are displayed in four systems (see Figures 6C–F). In both open and closed-state systems, the binding of inhibitor PF-0684003 resulted in a contraction of the channel located near the active site (1.2 nm from the channel entrance). It is noteworthy that the radius of the contracted channel was maintained at 0.17 nm, which is still higher than the molecular radius of the O_2_ (0.12 nm) and the H_2_O (0.14 nm), speculating that the inhibitor could only reduce the H_2_O/O_2_ passage efficiency rather than blocking it completely. After open-state binding of PF-06840003, it triggered the JK-Loop to flip inward, preventing the substrate L-Trp from entering the active site accompanied by small-molecule channel constriction, hindering O_2_/H_2_O entry. According to the axial distribution of the residues closest to the channel demonstrated in Figure 6B, the approaching residues were highly similar for the four systems. The common residues from the channel entrance to the active site are Q271, L292, G265, and A264, where both A264 and G265 are located on highly conserved loops (A260-A265), and A264 also has a stabilizing hydrogen bond with the inhibitor PF-06840003. In summary, for both open and closed states, inhibitor binding does not lead to substantial motor conformational changes in the O_2_/H_2_O channel, showing strong structural conservatism; and the highly conserved minor flexible loops (A260-A265) may be the valves determining whether the small molecules can reach the active site smoothly.

### 3.7 IDO1 signal transduction varies between open and closed states and is further attenuated by PF-06840003 binding

**FIGURE 7:**
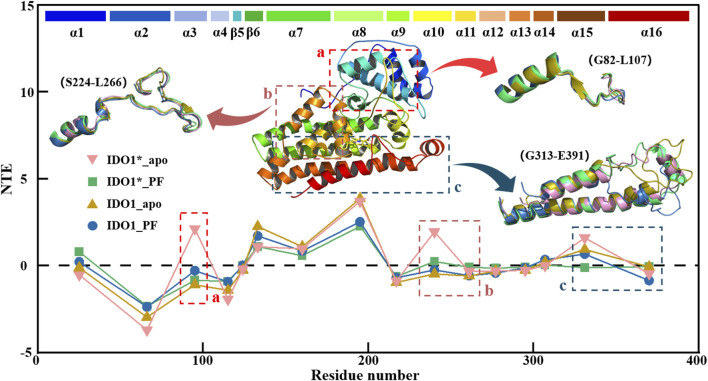
Transfer entropy (TE) analysis of IDO1 signal transduction in four conformational and binding states.

Figure 7 illustrates the global signal transduction of IDO1 across the four systems. In the closed state without inhibitor, IDO1 efficiently binds the substrate, with strengthened domain-residue coupling that establishes stable long-range pathways, resulting in the highest global NTE from the heme to distal functional sites. Binding of PF-0684003 in the closed conformation blocks or locks critical pathways, yielding the weakest global signal transmission. In the open state, coupling is weaker and pathways more dispersed than in the closed state, giving rise to moderate transmission, while PF binding in the open state perturbs local coupling but fails to fully lock the conformation, leading to reduced yet not minimal NTE. Overall, the signaling capacity follows the order: IDO1*_apo > IDO1_apo > IDO1_PF > IDO1*_PF. Region a, near the substrate channel, acts as a signal source in the closed apo state, where dynamic fluctuations help maintain structural stability, but this role is diminished in the open state or upon PF binding, likely due to local stabilization by the inhibitor. Region b, overlapping with the catalytic site, normally functions as a hub transmitting conformational information toward the heme, but this hub is disrupted by either PF binding or the open-state transition, leading to decreased NTE. Region c, which contains the JK-loop, cooperates with the heme and resdues such as His346 in the closed apo state to transmit C-terminal signals throughout the protein, but upon PF binding the inhibitor may interact with heme-adjacent residues or alter the electronic environment of Heme, effectively locking the JK-loop or restricting it to local coupling, thereby weakening signal propagation to distal regions.

### 3.8 A possible inhibitory mechanism of PF-0684003 against IDO1

**FIGURE 8:**
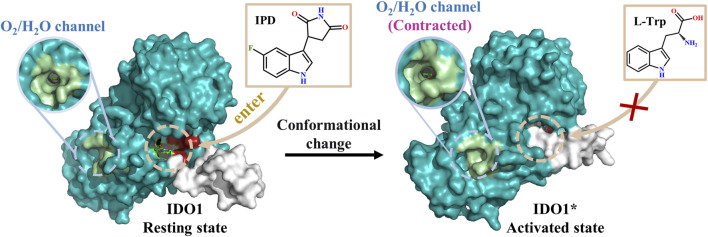
A possible inhibitory mechanism of PF-06840003 against IDO1.

Based on MD simulations and structural analyses, this study proposes a dual inhibitory mechanism of PF-06840003 against IDO1 (refer to Figure 8). In the open state, stretching of the JK-loop exposes the substrate channel, allowing PF-06840003 to enter pocket A and, through an N133-C129 hydrogen bond, draw the large and small domains closer to reduce pocket accessibility. Thus, even before full JK-loop closure, L-Trp binding is hindered, adding a site-competitive component to the non-competitive inhibition. Upon binding, PF-06840003 establishes extensive hydrogen bonds with surrounding residues, promotes inward flipping of the JK-loop, and shifts the protein toward the closed state. In the IDO1*_PF system, T379 of the conserved GTGG motif forms a stable hydrogen bond with the inhibitor, sealing off substrate channels and blocking L-Trp entry. Lewis-Ballester’s research shows that while both L-Trp and PF-06840003 insert their indole rings into pocket A, only amino side chain of L-Trp extends into pocket B to stabilize the active conformation (Lewis-Ballester et al., 2017). By contrast, succinimide ring of PF-06840003 fails to enter pocket B but compensates by forming stronger hydrogen bonds with T379 and an exclusive indol-S167 interaction. Collectively, PF-06840003 inhibits IDO1 not only by conformational regulation *via* JK-loop closure but also through competitive occupation of pocket A, jointly preventing productive L-Trp binding and disrupting the kynurenine pathway.

### 3.9 Molecular design of indolepyrrodione inhibitors

#### 3.9.1 Establishment of 3D-QSAR models

In recent years, numerous IDO1 reaction pathways and inhibition mechanisms have been reported, and indolepyrrodione (IPD) inhibitors have also been widely reported. In fact, the establishment of three-dimensional quantitative conformational relationships between a series of IPD inhibitors based on their structure and activity data is of great theoretical and practical value for the optimization of these inhibitors. As illustrated in Supplementary Figure S7, considering the shared core structure and the identical binding mechanism in the active site of IDO1 for L-Trp and PF-06840003: the indole rings are positioned within pocket A and are oriented perpendicular to heme (Lewis-Ballester et al., 2017). In this work, a total of 26 small molecules of IPD inhibitors were collected, of which Comp #15-21 were L-Trp analogues (Macchiarulo et al., 2009) and the rest were PF-06840003 analogues. In the present work, a total of 26 small molecules of IPD inhibitors were collected (Crosignani et al., 2017).

**FIGURE 9:**
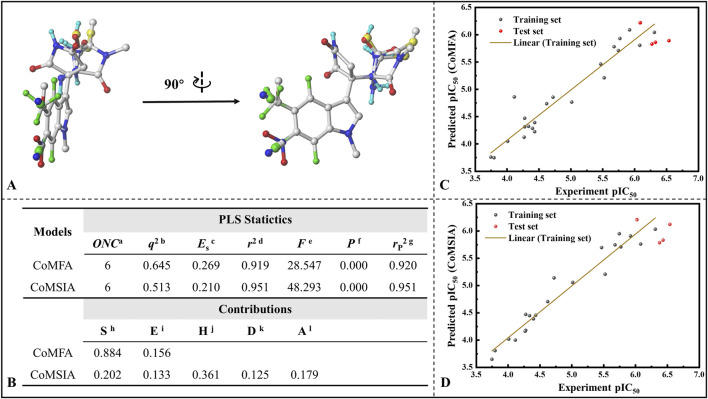
Establishment of 3D-QSAR models for indolepyrrodione inhibitors against IDO1. **(A)** Molecular structural superimposition; **(B)** Various statistical parameters of the CoMFA and CoMSIA models; Correlation between predicted pIC_50_ values and experimental values based on the CoMFA model **(C)** and CoMSIA model **(D)** Acronym primarily depicted in Figure **(B)** ^a^Optimum number of components. ^b^Leave-one-out (LOO) cross-validated correlation coefficient. ^c^Standard error of estimate. ^d^Noncross-validated correlation coefficient. ^e^F-Test value. ^f^Probability of *r* ^2^. ^g^Predicted correlation coefficient for the test set. ^h^Steric field. ^i^Electrostatic field. ^j^Hydrophobic field. ^k^H-bond donor field. ^l^H-bond acceptor field.

Subsequently, all 26 molecules were aligned, and it can be seen from Figure 9A that the structural alignment of all the small molecules was very good, with the indole ring parts highly overlapping, which laid the foundation for the rationality of 3D-QSAR modeling. In three-dimensional quantitative structure-activity relationship (3D-QSAR) studies, the division of training and test sets represents a crucial step in developing robust and predictive models. The primary objective of this partitioning is to ensure that the training set adequately captures the chemical diversity and activity range of the compounds to be predicted, thereby enabling the model to reliably extrapolate to the test set, which simulates novel molecules. Therefore, rigorous validation through external or cross-validation is essential to assess model reliability. To this end, we conducted statistical comparisons of key molecular descriptors between the training and test sets, confirming the absence of significant differences in molecular weight (MW), number of hydrogen bond donors (nHBD), molecular surface area (MSA), and molecular solubility (MS) (t-test p > 0.05) (refer to Supplementary Figure S8).On this basis, 22 molecules were randomly screened as the training set (1-22) of the 3D-QSAR model, and the remaining 4 molecules as the test set (23-26). Figure 9B lists the various statistical parameters of the CoMFA and CoMSIA models. In the CoMFA model, the cross-validated correlation coefficient (q2) and non-cross-validated correlation coefficient (r2) were 0.645 and 0.919, respectively. Meanwhile, the F value was 28.547 and the activity prediction correlation coefficient was 0.920, which indicated that the CoMFA model had a good prediction ability. In terms of the contribution rate, the steric field (S) of the CoMFA model was 88.4%, and the electrostatic field (E) was 15.6%, indicating that the steric field greatly affected the inhibitory activity of the inhibitor. The cross-validated correlation coefficient, non-cross-validated correlation coefficient and F value of the CoMSIA model were 0.513, 0.951 and 48.293, respectively, and the correlation coefficient of activity prediction was 0.951. The contributions of steric field (S), electrostatic field (E), hydrophobic field (H), hydrogen bond donor field (D) and hydrogen bond acceptor field (A) were 20.2%, 13.3%, 36.1%, 12.5% and 17.9%. It is not difficult to find that the hydrophobic field also has a large influence on its bioinhibitory activity. Figure 9C and D give the correlation between the predicted pIC50 and the experimental values based on the CoMFA and CoMSIA models, with correlation coefficients of 0.919 and 0.951, respectively. Excellent linear correlation suggests that both models have a good ability of activity prediction, and can be compared to and complemented with each other. Based on the results of CoMFA and CoMSIA models, it was inferred that the biological activity of IPD inhibitors could be improved mainly by changing the molecular size and hydrophobicity.

The detailed inhibition activity prediction results for both the training and test sets in the CoMFA and CoMSIA models are listed in Supplementary Table S3. Combined with Figures 9C,D mentioned above, all deviations (Res.) between the predicted values (Pred.) and experimental values (pIC_50_) are less than 1, while the error rates remain below 15% for both CoMFA and CoMSIA models. Notably, some individual predictions, such as Cmpd #17, exhibit almost identical values to the experimental ones, indicating that both models possess strong predictive capabilities. The stable and predictive performance of these 3D-QSAR models makes them useful for evaluating IPD inhibitors and guiding rational design.

**FIGURE 10:**
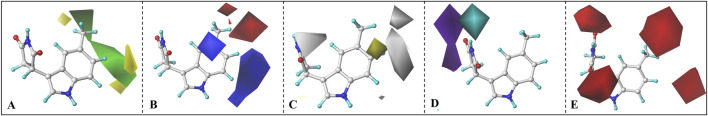
CoMFA and CoMSIA contour maps. **(A)** Steric field, **(B)** electrostatic field, **(C)** Hydrophobic field, **(D)** H-bond donor field, **(E)** H-bond acceptor field.

Taking Cmpd #5 as an example, Figure 10 llustrates the contour maps of CoMFA and CoMSIA models, with a truncation ratio of 80%: 20%. Based on the three-dimensional field distribution of CoMFA (refer to Figure 10A), Cmpd #5 exhibits three yellow and two green blocks on one side of the indole ring. The yellow blocks are positioned at both ends of the central benzene ring in the indole, indicating that introducing bulky groups in this region is unfavorable for enhancing biological activity. This elucidates why Cmpd #23 demonstrates higher inhibitory potency compared to Cmpd #14, which has a methyl group introduced at the ortho position (refer to Supplementary Figure S7). In terms of the electrostatic field contour map, two red and two blue blocks enclose the indole ring (see Figure 10B). The red blocks primarily exist between positions 5 and 6 of the indole ring, while blue blocks predominantly surround positions 4 and 7. From an electrostatic perspective, red blocks signify that incorporating negatively charged groups at these positions enhances inhibitory activity, whereas blue blocks suggest that only positive charges are favorable for increasing inhibition. This also clarifies why compounds #12, #13, #23, and #24 exhibit higher activity than compound #1 and emphasizes that introducing strongly electronegative groups at position 5 is more advantageous.

Compared to the CoMFA model, the CoMSIA model provides additional insights into hydrophobic and hydrogen bond donor/acceptor fields (refer to Figures 10C–E). In Figure 10C, a gray block surrounds the entire benzene ring of indole, indicating that introducing hydrophobic groups in this region would be unfavorable for enhancing molecular inhibitory activity. The potential maps for hydrogen bond donor/acceptor fields display cyan/purple blocks representing favorable/unfavorable regions for hydrogen bond donors and red blocks representing favorable regions for hydrogen bond acceptors. To optimize inhibitor activity, it is advantageous to incorporate groups containing hydrogen bond donors (such as hydroxyl or carboxyl groups) in the cyan block region or introduce structures with hydrogen bond acceptors in the red region. In other words, connecting a hydrogen bond donor group to the nitrogen atom on the succinimide attached to the indole ring benefits compound activity, while incorporating a hydrogen bond acceptor at positions 5 and 6 of the indole ring is more advantageous.

In conclusion, for IPD inhibitors, a CoMFA and CoMSIA model with excellent predictive capability was developed. Based on the contour maps derived from the 3D-QSAR analysis, several valuable insights are provided to enhance inhibitor activity. For instance, the introduction of hydrogen bond acceptors with smaller volume and highly electronegative at positions 5 and 6 of the indole ring; the addition of electron-donating groups at positions 4 and 7; or induction of substituents containing hydrogen bond donors at the nitrogen atom of the succinimide ring, *etc.*

#### 3.9.2 Drug design guided by binding free energy

In the field of computer-aided drug design (CADD) based on molecular structure, a widely adopted strategy is to select potential compounds with high activity by predicting the minimum binding free energy (Huang and Jacobson, 2010; Rapp et al., 2011). Using three binding free energy calculations (Robetta, MM/PBSA, and SIE), Cui and his team predicted thermally mutated residues of P53 and identified molecules that potentially inhibit mitogen-activated protein kinase (MAPK) signaling and possess anticancer properties (Cui et al., 2008). Similarly, Shen et al. in the study of New Delhi metallo-β-lactamase-1 (NDM-1) inhibitors published a mercapto inhibitor exhibiting high activity using enzyme activity assays and MM/PBSA binding free energy calculations (Shen et al., 2017). Elkarhat et al. used molecular docking, molecular dynamics simulations and MM-PBSA analysis to screen two potential inhibitors in the study of SARS-cov-2 RNA-dependent polymerase (nsp12) inhibitors (Elkarhat et al., 2022). Statistically, over 25% of drug molecules contain halogen atoms, and the bonding energy of halogen bonds increases with electronegativity (Li et al., 2012). Among them, fluorine has a unique electron attraction and its strong electronegativity may lead to the formation of stronger interactions with bonds between neighboring atoms, which is widely regarded as a superior strategy. Duan et al. performed halogen substitution of the uracil C5 atom in a recognition and release study between uracil and hCNT3, and the results of the SIE coupled with free energy calculations showed fluorine to be the optimal alternative (Duan et al., 2022). In addition, hydrogen bond donor introduction is also used as a common strategy for lead compound modification, Peng et al. found that replacing the hydrogen bond acceptor (carbonyl) with a hydrogen bond donor (hydroxyl) greatly improved IDO1 inhibitor activity (Peng et al., 2016).

**FIGURE 11:**
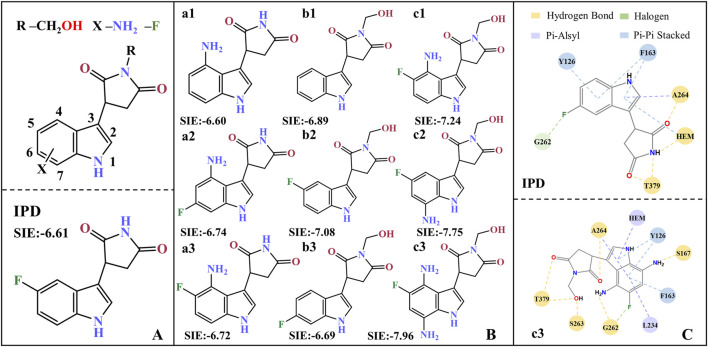
Molecular design of IPD inhibitors guided by binding free energy. **(A)** Molecular scaffolds; **(B)** 9 potentials highly active IPD inhibitors and experimental data of inhibitors; **(C)** Contact residues and interactions between PF-06840003 inhibitor and c3 molecule within a 0.4 nm range. The units for SIE free energy data are kcal·mol^−1^.

Based on the molecular modification suggestions given by CoMFA and CoMSIA models, a series of IPD inhibitors were designed by fluorine substitution and hydrogen bond donor introduction. Firstly, nine small molecules showed the potential to have superior inhibitory activity by SIE free energy calculations (see Figure 11). Based on the inhibitor PF-06840003, the introduction of -NH_2_ at position 4, as well as electronegative fluorine groups at positions 5 and 6 of the indole ring, respectively, yielded a1, a2, and a3. By the calculations, the binding free energies of a2 and a3 were −6.74 and −6.72 kcal·mol^−1^, respectively, with a slight decrease compared to −6.60 for the inhibitor a1. Based on the introduction of hydrogen bond donor groups on the ring nitrogen of the inhibitor succinimide, compounds b1-b3 were designed in order to explore the difference between the introduction of small site resistance strongly electronegative groups at positions 5 and 6 of the indole ring. It can be seen that the introduction of the group at position 5 showed better improvement in the inhibitor activity. In addition, b2 showed greater improvement over the inhibitor PF-06840003 activity, which may be due to the fact that hydrogen bonding is essential for the stable binding of the inhibitor in the active site.

Combining fluorine substitution and hydrogen bond donor introduction strategies, compounds c1-c3 were designed. Among them, c3 has the lowest binding free energy of −7.96 kcal/mol among the designed molecules, making it a promising potential seedling molecule in theory, but its actual inhibitory activity against IDO1 needs to be verified by *in vitro* enzyme activity experiments and cell-based assays (see Figure 11B). Comparing all the residues within 4 Å around the active site between the inhibitor PF-06840003 and compound c3, the former has only six contacting residues; whereas c3 possesses nine contacting residues and binds more tightly (see Figure 11C). In the c3 molecule, the introduction of an amino group in the indole ring allows the formation of a hydrogen bond with S167/G262; the adoption of fluorine allows the formation of a halogen bond with G262; and the succinimides nitrogen inducer hydroxymethyl forms a hydrogen bond with T379/S263. In addition, c3 forms π-alkyl interactions with Heme/L234/A264 and π-π interactions with Y126/F163. It is noteworthy that G262/S263/A264/Y126/F163 is located in the active pocket A (Coletti et al., 2017) of inhibitor binding, T379 is essential for the formation of a closed conformation in the JK-Loop region to ensure stable binding of the inhibitor (Álvarez et al., 2016), S167 plays an important role in the inhibitory activity of a variety of IDO1 inhibitors (Tomek et al., 2017), and L234 is essential for the catalytic activity of IDO1 (Liu et al., 2020).

It is worth noting that a lower binding free energy prediction holds significant importance as an essential parameter for potential lead compounds. Moreover, comprehensive consideration should be given to pharmacokinetic parameters encompassing absorption, distribution, metabolism, and excretion. While CADD plays a pivotal role in lead compound development, it is equally imperative to conduct pharmacokinetic experiments, toxicity safety assessments, and tolerance tests. Therefore, upon completion of the drug design process, a series of subsequent tasks need to be undertaken. For instance, the utilization of MTT colorimetric assay enables the comprehensive evaluation of lead compound toxicity on cells to assess their impact thoroughly; high-throughput assessment of compound-target protein interactions can be achieved using the Fluorescence Polarization (FP) method; observation of radioactive substrate conversion or changes in enzyme activity through radioligand inhibition assays facilitates the evaluation of drugs’ inhibitory effects on enzymes.

## 4 Conclusion

In this paper, a series of simulations were used to compare the molecular recognition, conformational changes and movement characteristics of PF-06840003 with IDO1 in both open and closed states. Subsequently, 3D-QSAR modeling of IPD-like inhibitors targeting IDO1 is developed, which ultimately gives novel seedling molecules with potentially high activity using the lowest free energy-directed strategy. In this work, two conformations of IDO1 open state and IDO1* closed state were firstly modeled and validated for their plausibility. Based on comparative MD trajectories of IDO1_apo, IDO1*_apo, IDO1_PF and IDO1*_PF, IDO1_apo open state stability is higher than that of IDO1*_apo in the absence of inhibitor binding. The binding of PF-06840003 leads to the formation of an extensive hydrogen bonding network, which can result in an inversion of the JK-Loop region and a tendency for the system to favor the closed state (i.e., IDO1* _PF). In the IDO1* _PF closed complex system, the substrate entry/exit channels are capped both to prevent leakage of the inhibitor and to block the entry of L-Trp. In addition, the small molecule channel (O_2_/H_2_O) shrinks and narrows, and the efficiency of molecular access to the active site decreases. Moreover, transfer entropy analysis revealed the key directional information flow between the JK-loop and the active site, suggesting that inhibitor binding reshapes the dynamic communication pathways essential for catalysis. In the second part, this paper also establishes a CoMFA and CoMSIA model with good activity prediction ability for IPD inhibitors and gives some suggestions that may help to enhance the activity of the inhibitors. Based on the IDO1 structure and its interaction with IPD inhibitors, nine derivative molecules were designed, among which C3 was identified as the most promising lead candidate based on its lowest predicted binding free energy; however, its inhibitory activity still requires experimental validation. In conclusion, the present work not only reveals the molecular recognition and mechanism of action of IPD inhibitors with IDO1 at the atomic level but also provides feasible optimization strategies that serve as important references for the subsequent design and development of novel IDO1 inhibitors. Future studies should further validate the actual inhibitory activity of the candidate molecules through *in vitro* assays, and conduct pharmacokinetic and toxicity evaluations to promote the translation of theoretical designs into practical applications.

## Data Availability

The original contributions presented in the study are included in the article/Supplementary Material, further inquiries can be directed to the corresponding authors.
