# Supplementary material for: Action mechanism and molecular design of indolepyrrodione inhibitors targeting IDO1
Source: Front Mol Biosci. 2025 Oct 23;12:1661700. doi: 10.3389/fmolb.2025.1661700 (PMC12588868; doi:10.3389/fmolb.2025.1661700)
Supplement: Supplementary file 1 [file Supplementaryfile1.docx]

**Action Mechanism and Molecular Design of Indolepyrrodione Inhibitors Targeting IDO1**

Xinmin Wang^1, †^, Zhigang Zhang ^2, †^, Kaixuan Hu ^2, †^,Wentong Yu ^2^, Yan Cheng ^1^, Yuting Song ^1^, Xin Sun ^2^, Siyao Li ^2^, Tiantian Yang ^2^, Jianping Hu ^2^, Jing Jing ^1,^ *,Ting Luo ^1,^ *

^1^ Laboratory of Integrative Medicine, Clinical Research Center for Breast, State Key Laboratory of Biotherapy, West China Hospital, Sichuan University and Collaborative Innovation Center, Chengdu, Sichuan 610041, China.

2 Key Laboratory of Medicinal and Edible Plants Resources Development of Sichuan Education Department, School of Pharmacy, Chengdu University, Chengdu, China.

^†^ These authors contributed equally to this work.

^*^ Correspondence: jingjing@wchscu.cn(J.J.); luotingwch@163.com (T.L)


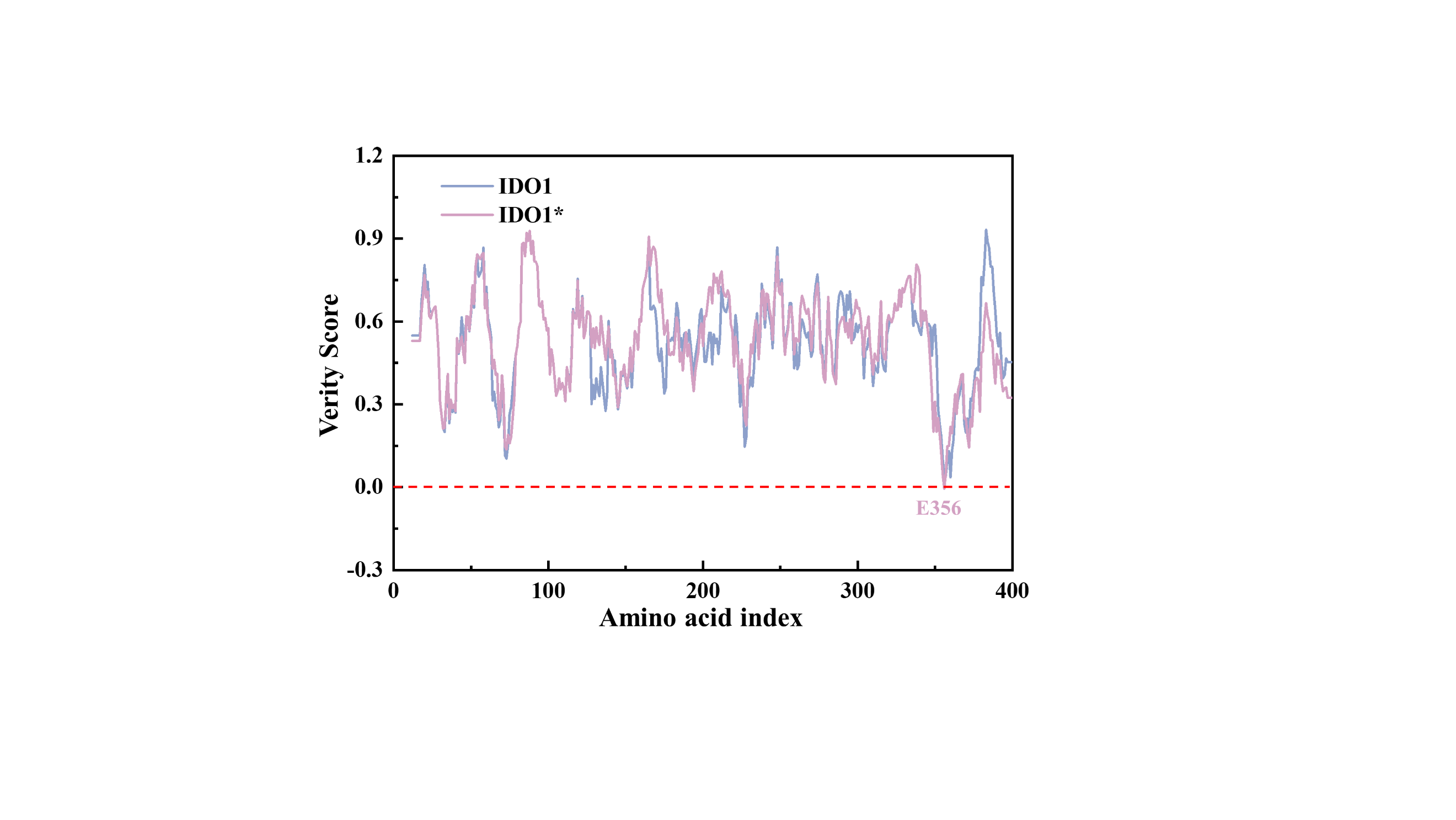


**Figure S1.** Distribution of Verify Score at the amino acid level for the full-length models of IDO1 (blue) and IDO1* (red).


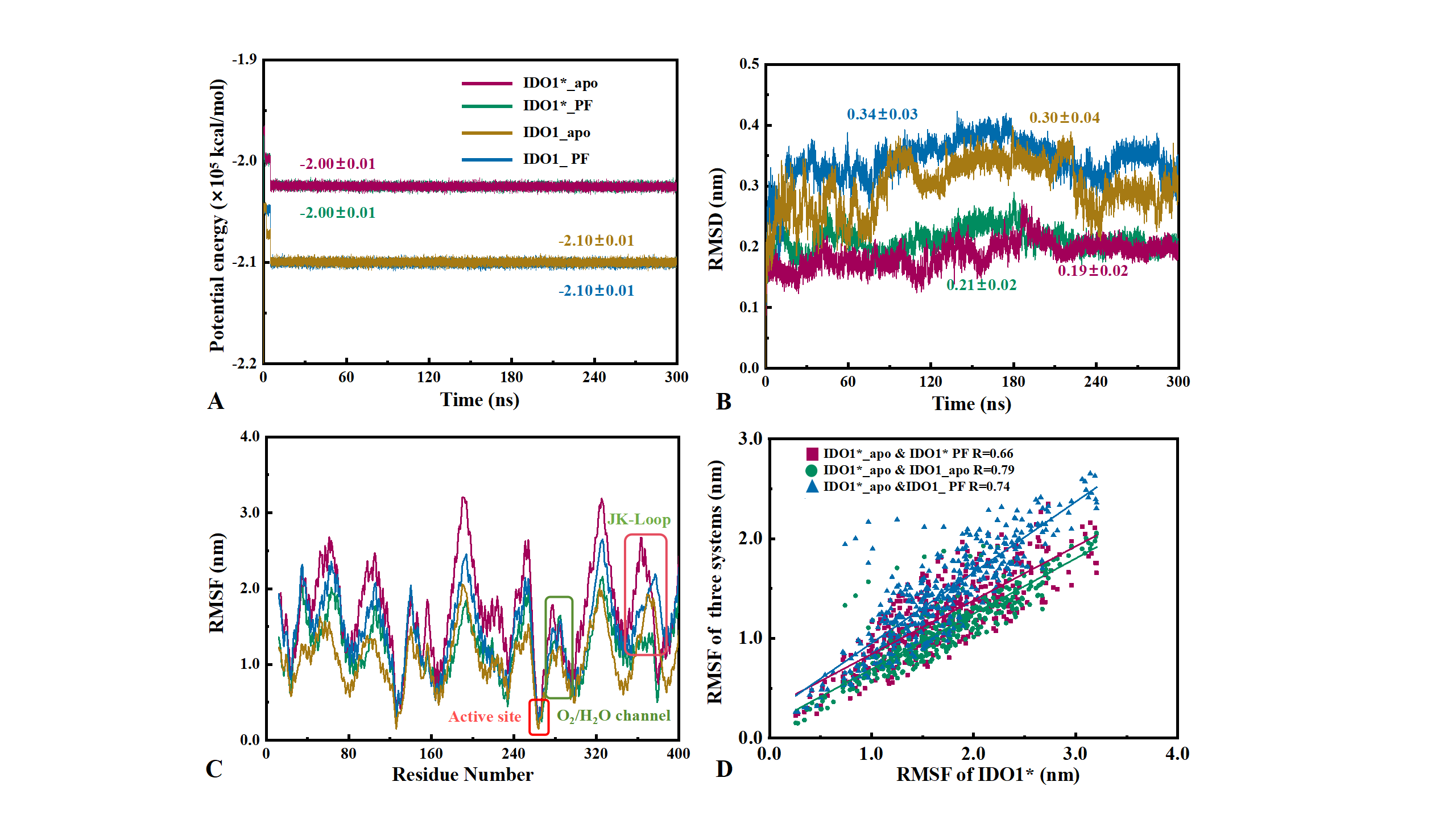


**Figure S2**. Convergence characteristics of the MD trajectories for four systems, IDO1_apo, IDO1*_apo, IDO1_PF and IDO1*_PF. Variation of potential energy (A) and RMSD (B) over time; distribution of RMSF at the residue level (C) and its correlation analysis (D) The red/yellow/green boxes are used to represent the small flexible loop around the active site (A260-A265), the small molecule channel for O_2_/H_2_O (S263-S312), and JK-Loop (Q360-D383).


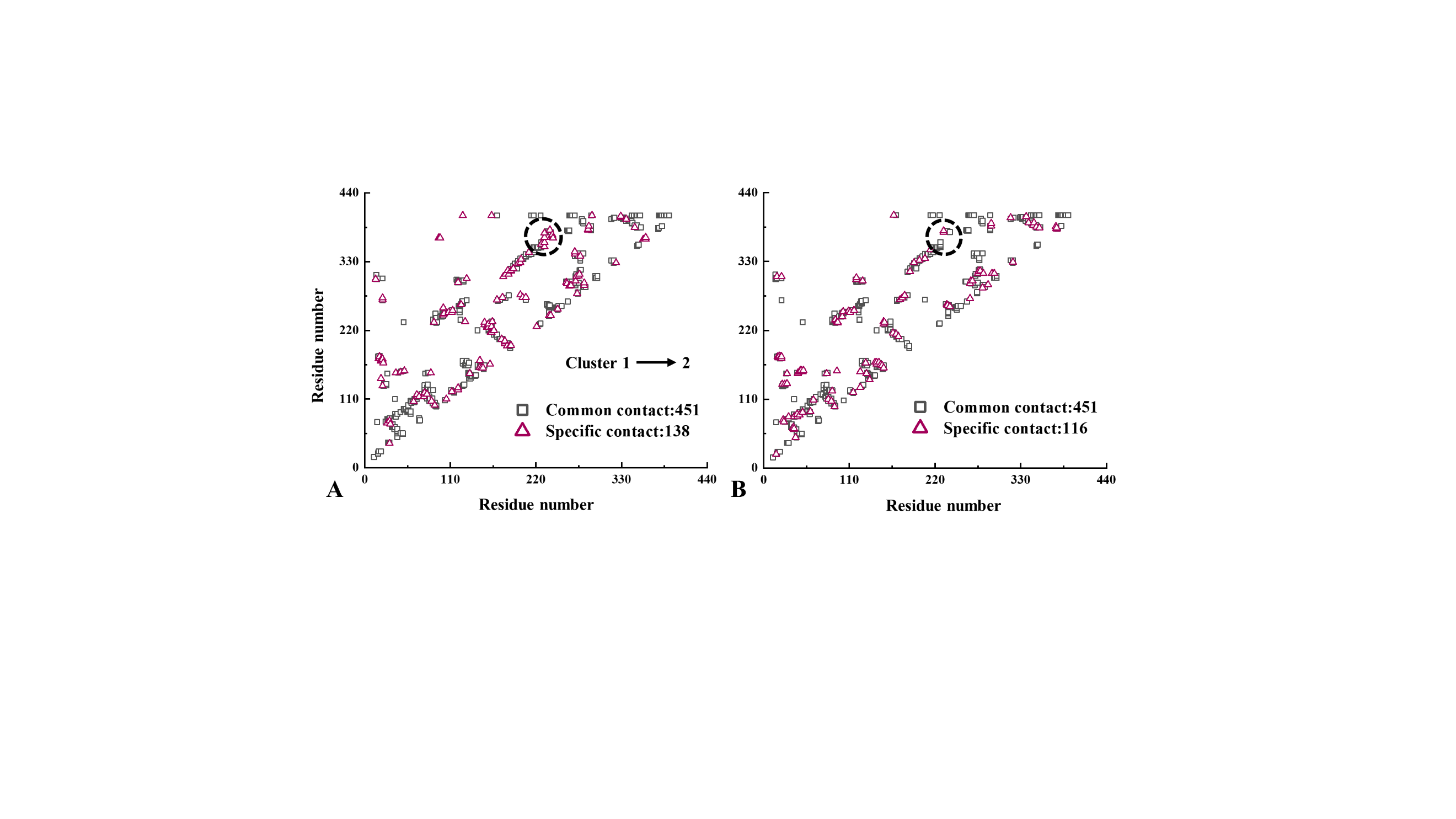


**Figure S3**. Comparison of contact residues between the representative conformations of the first cluster (A) and the second cluster (B) in the IDO1*_apo system. The black dashed box highlights the main differences in contact residues, namely JK-Loop (Q360-D383) and residues near the active site H220-E250.


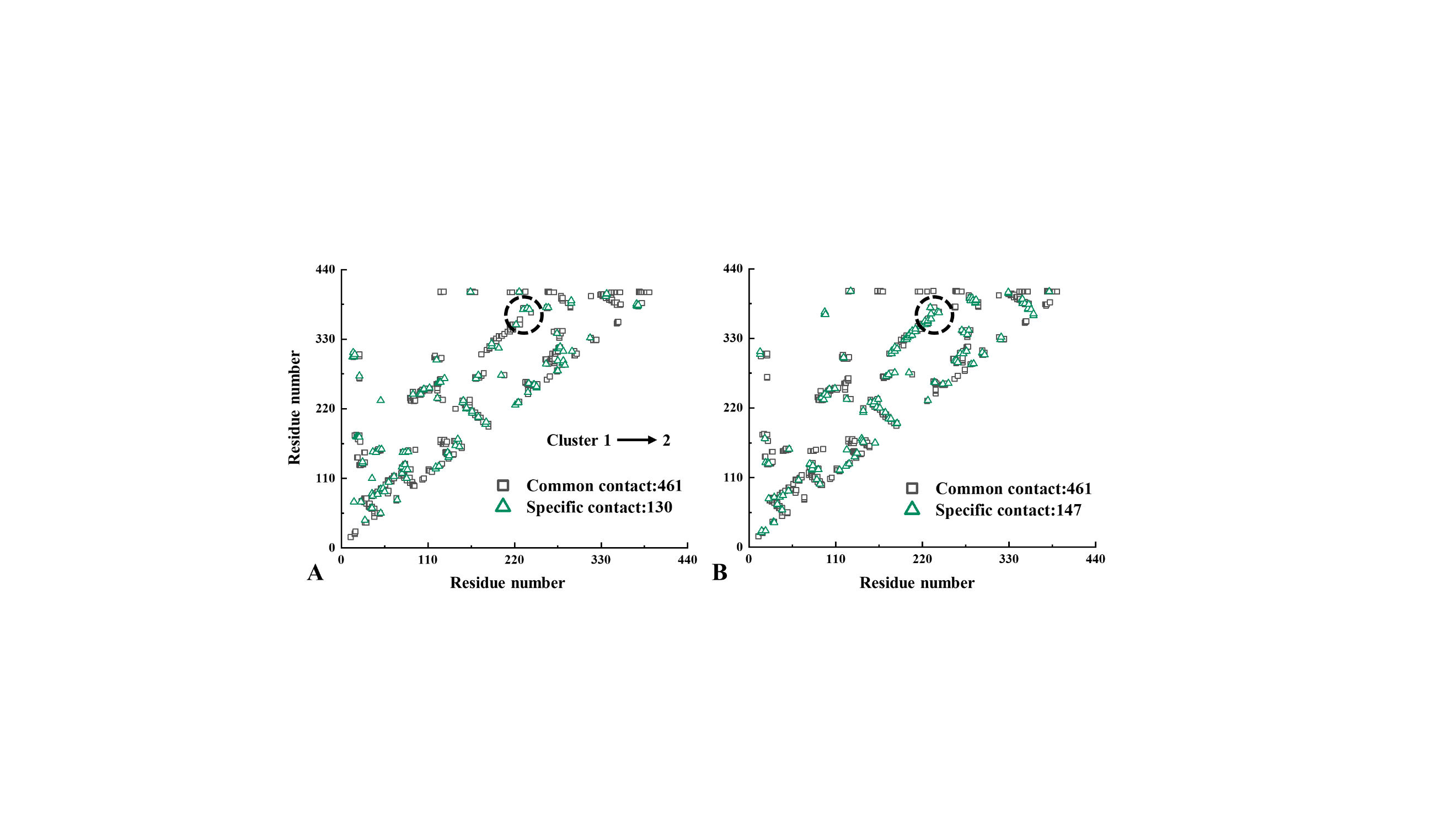


**Figure S4.** Comparison of contact residues between the representative conformations of the first cluster (A) and the second cluster (B) in the IDO1*_PF system. The black dashed box highlights the main differences in contact residues, namely JK-Loop (Q360-D383) and residues near the active site H220-E250.
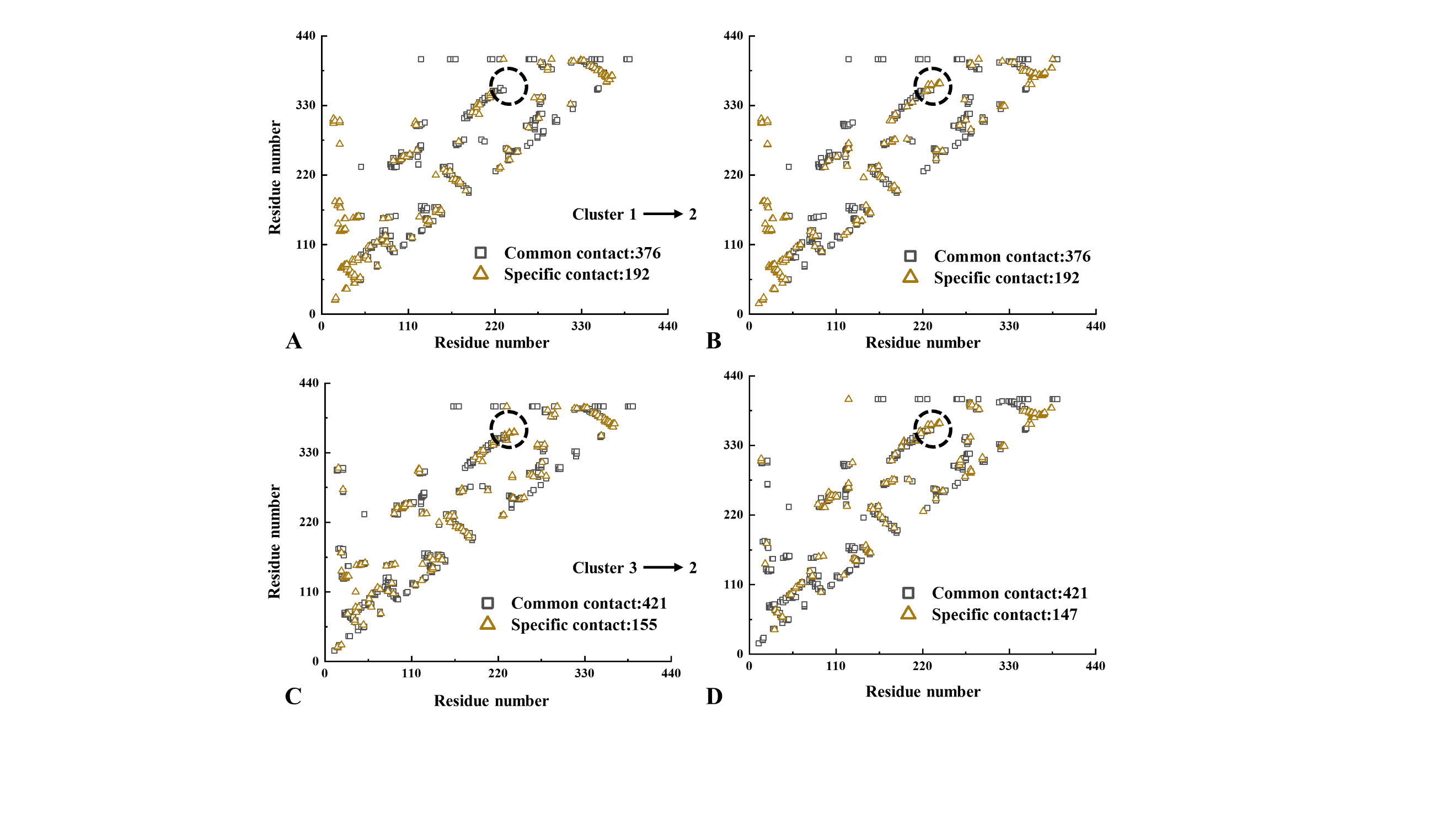


**Figure S5.** Comparison of contact residues between representative conformations of the first cluster. (A), second clusters (B, D) and third cluster (C) in the IDO1_apo system. The black dashed box highlights the main differences in contact residues, namely JK-Loop (Q360-D383) and residues near the active site H220-E250.


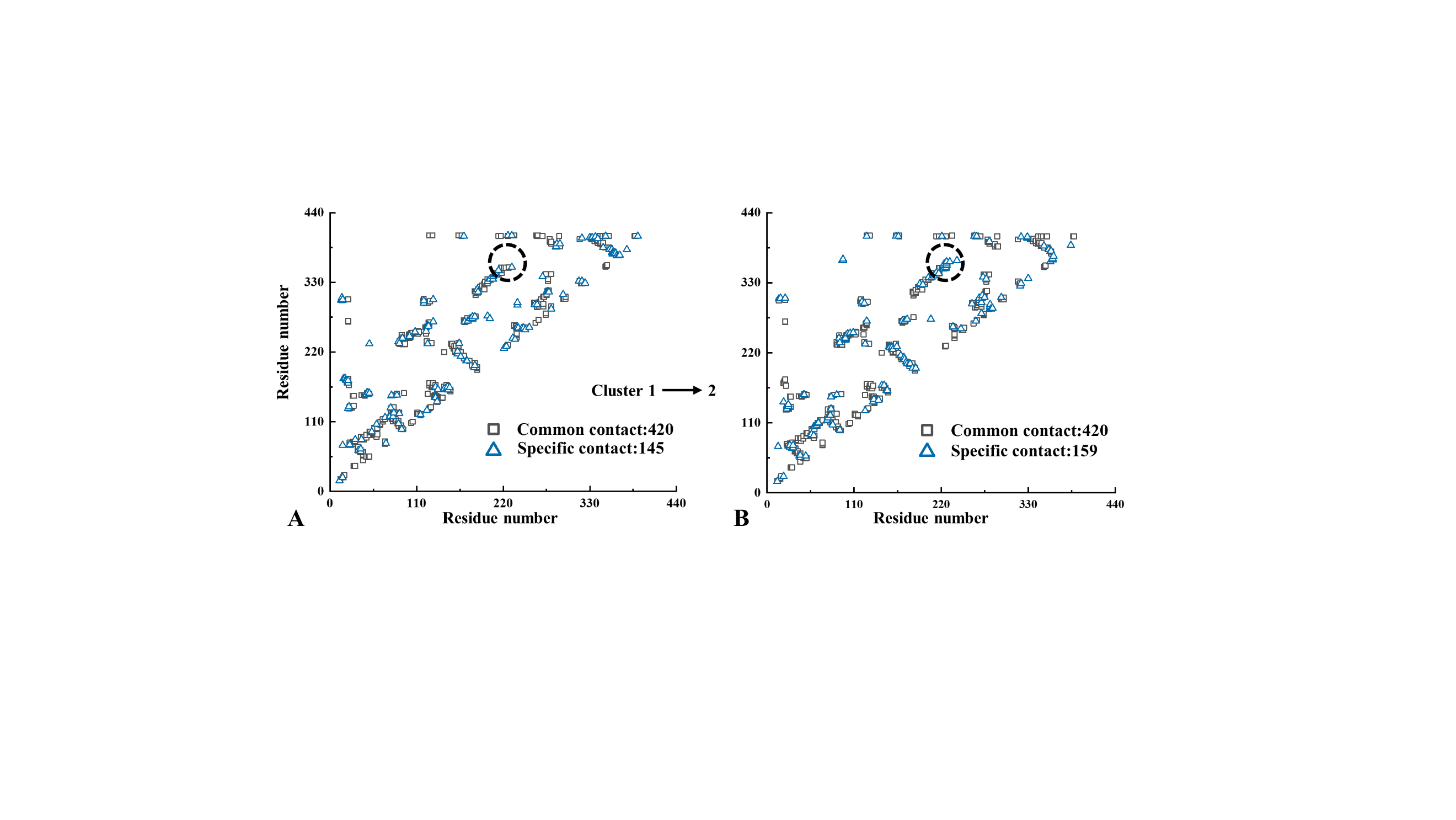


**Figure S6.** Comparison of contact residues between the representative conformations of the first cluster (A) and the second cluster (B) in the IDO1_PF system. The black dashed box highlights the main differences in contact residues, namely JK-Loop (Q360-D383) and residues near the active site H220-E250.


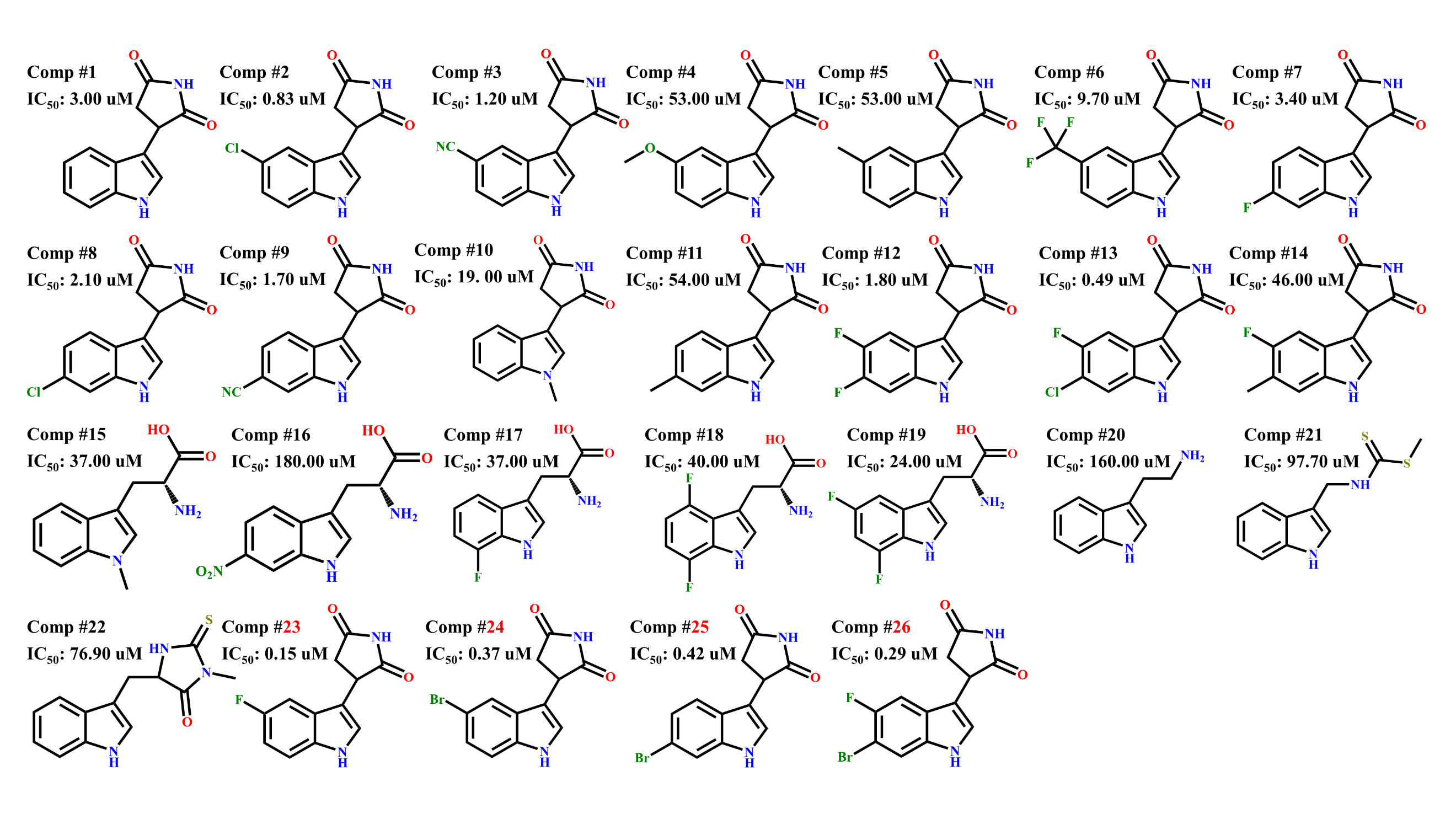


**Figure S7**. Structures of 26 IPD analogue inhibitors used for constructing a 3D-QSAR model.


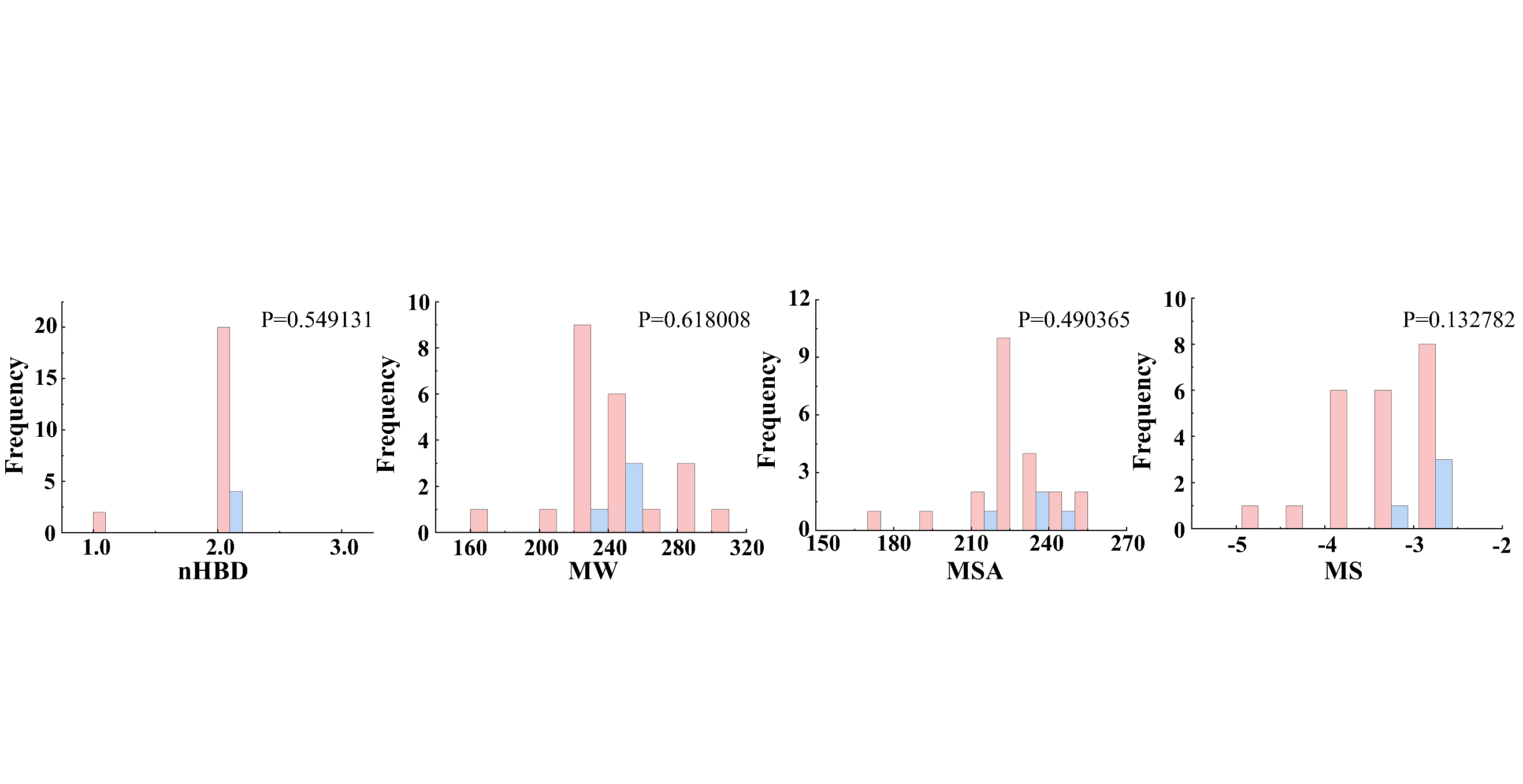


**Figure S8**. Distribution of 4 molecular properties of dipeptide compounds. Includes nHBD, MW, MSA and MS.

**Table S1**. Currently available bioinformatics data for IDO1 structures.

| **PDB IDs** | **Small Molecules** | **Mut.** | **Resolution (Å)** | **PDB IDs** | **Small Molecules** | **Mut.** | **Resolution (Å)** |
| --- | --- | --- | --- | --- | --- | --- | --- |
| 2D0T^[72]^ | HEM,NHE,PIM | 0 | 2.3 | 6V52^[84]^ | QPV | 0 | 1.78 |
| 2D0U^[72]^ | CYN,HEM,NHE | 0 | 3.4 | 6WJY^[85]^ | U41 | 0 | 1.91 |
| 4PK5^[73]^ | HEM,PKJ | 0 | 2.79 | 7A62^[86]^ | CL,GOL,HEM | 2 | 2.44 |
| 4PK6^[73]^ | HEM, PKL | 0 | 3.45 | 6UBP^[87]^ | CMO,HEM,TRP | 0 | 2.95 |
| 4U72 | HEM,NHE,PIM | 1 | 2.0 | 7AH4^[88]^ | HEM,RCN | 0 | 2.40 |
| 4U74 | HEM,NHE,PIM | 1 | 2.31 | 7AH5^[88]^ | HEM,RCQ | 0 | 2.9 |
| 5EK2^[74]^ | 5PJ, HEM | 0 | 2.68 | 7AH6^[88]^ | GOL,HEM,RCW | 0 | 3.00 |
| 5EK3^[74]^ | 5PK, HEM | 0 | 2.21 | 6WPE^[89]^ | U6G | 0 | 2.43 |
| 5EK4^[74]^ | 5PF , HEM | 0 | 2.64 | 6X5Y | URJ | 0 | 2.65 |
| 5ETW^[74]^ | HEM, XNL | 0 | 2.7 | 7E0O^[90]^ | HEM,HS0 | 0 | 3.34 |
| 5XE1^[75]^ | HEM,IUU | 0 | 3.2 | 7E0P^[90]^ | ACY,HEM,HU0 | 0 | 2.64 |
| 5WMU^[76]^ | CYN,HEM,TRP | 0 | 2.4 | 7E0Q^[90]^ | HEM,HU3 | 0 | 2.46 |
| 5WMV^[76]^ | CYN,HEM,TRP,ZCW | 0 | 2.6 | 7E0S^[90]^ | ACY,HEM,HU6 | 0 | 2.71 |
| 5WMW^[76]^ | CYN, HEM, TRP | 1 | 3.03 | 7E0T^[90]^ | HEM,HU9 | 0 | 2.13 |
| 5WMX^[76]^ | CYN,HEM,TRP ,ZCW | 1 | 2.69 | 7E0U^[90]^ | HEM,HUC | 0 | 2.28 |
| 5WN8^[76]^ | BBJ, HEM | 0 | 2.5 | 7M63^[91]^ | TRP | 0 | 3.1 |
| 5WHR^[77]^ | PF-06840003,HEM | 0 | 2.28 | 7M7D^[91]^ | HEM,YRM | 0 | 2.6 |
| 6F0A^[78]^ | ALA,C82,HEM | 0 | 2.26 | 6R63^[92]^ | HEM,JTP | 0 | 2.90 |
| 6AZU^[79]^ | HEM, SO4 | 0 | 2.82 | 6PU7^[93]^ | HEM,OY4 | 0 | 2.43 |
| 6AZV^[79]^ | C4V | 0 | 2.76 | 6PZ1^[94]^ | HEM,PF-06840003,GOL | 0 | 2.65 |
| 6AZW^[79]^ | C51 | 0 | 2.78 | 6KOF^[95]^ | DO9,HEM | 0 | 2.26 |
| 6CXU^[80]^ | CYN,HEM,TRP | 1 | 2.49 | 6KPS^[95]^ | DU6,HEM | 0 | 2.45 |
| 6CXV^[80]^ | CYN,HEM,TRP ,ZCW | 1 | 2.6 | 6KW7^[95]^ | DYC,HEM | 0 | 3.02 |
| 6E35 | CYN,HEM,TRP ,ZCW | 0 | 2.41 | 7B1O^[91]^ | SLW | 0 | 2.58 |
| 6DPQ^[81]^ | GOL,H7P,HEM | 0 | 2.94 | 7NGE^[96]^ | CL,GOL,HEM,NA,NFK,PO4,TRP | 2 | 2.3 |
| 6DPR^[81]^ | EDO,H7P , HEM | 0 | 3.2 | 7P0N^[96]^ | CL,GOL,HEM,NFK,OXY,PO4,TRP | 2 | 2.5 |
| 6MQ6^[81]^ | GOL,H7P , HEM | 0 | 3.05 | 7P0R^[96]^ | GOL,HEM,NFK,PO4,TRP | 2 | 2.5 |
| 6E40^[82]^ | BBJ, HEM | 2 | 2.31 | 7RRB^[97]^ | 6ZI | 0 | 2.69 |
| 6E41^[82]^ | HEM, HQS | 2 | 2.29 | 7RRC^[97]^ | 6RI | 0 | 2.18 |
| 6E42^[82]^ | HEM, HQJ, PO4 | 2 | 2.1 | 7ZV3^[98]^ | GOL,HEM,RCT,SO4 | 0 | 2.55 |
| 6E43^[82]^ | BEZ, HQM | 2 | 1.71 | 7RRD | 6IZ | 0 | 2.76 |
| 6E44^[82]^ | HEM, PO4 | 2 | 1.9 | 8ABX^[99]^ | N2U,N39,PG4,PGE | 0 | 1.65 |
| 6E45^[82]^ | GOL, HEM, PO4 | 2 | 2.0 | 8I7L^[100]^ | OIH,THJ | 0 | 2.8 |
| 6E46^[82]^ | HEM, PO4, TRP | 2 | 2.09 | 7YXT | GOL,HEM,OXY | 2 | 2.48 |
| 6O3I^[83]^ | HEM,LKP | 0 | 2.69 | 7Z2L | GOL,HEM,KYN,OXY | 2 | 2.56 |

**Table S2**. Key residues recognized by IDO1 and IDO1* in the complex system with inhibitor PF-06840003 (kcal·mol-1).

| **Systems** | **Residues** | ***VDW*_MM_** | ***ELE*_MM_** | ***ELE*_GB_** | ***VDW*_SA_** | ***E*_tot_** |
| --- | --- | --- | --- | --- | --- | --- |
| **IDO1*_PF** | S263 | -1.08 ± 0.24 | -4.06 ± 0.96 | 2.25 ± 0.31 | -0.10 ± 0.02 | -2.99 ± 0.49 |
|  | A264 | -1.37 ± 0.29 | -2.11 ± 0.56 | 1.27 ± 0.16 | -0.16 ± 0.01 | -2.37 ± 0.14 |
|  | T379 | -1.50 ± 0.23 | -1.68 ± 0.41 | 1.29 ± 0.17 | -0.12 ± 0.01 | -2.01 ± 0.39 |
|  | S167 | -0.49 ± 0.13 | -2.32 ± 0.33 | 1.51 ± 0.01 | -0.06 ± 0.02 | -1.35 ± 0.21 |
|  | F163 | -1.58 ± 0.33 | -1.04 ± 0.28 | 1.80 ± 0.05 | -0.20 ± 0.03 | -1.02 ± 0.55 |
|  | Y126 | -1.26 ± 0.11 | -0.73 ± 0.06 | 1.10 ± 0.15 | -0.06 ± 0.01 | -0.95 ± 0.03 |
|  | L234 | -0.94 ± 0.13 | 0.03 ± 0.02 | 0.02 ± 0.01 | -0.04 ± 0.01 | -0.94 ± 0.16 |
|  | F226 | -0.77 ± 0.11 | -0.10 ± 0.09 | 0.27 ± 0.11 | -0.07 ± 0.01 | -0.67 ± 0.11 |
| **IDO1_PF** | S263 | -1.12 ± 0.24 | -3.22 ± 0.73 | 2.26 ± 0.29 | -0.14 ± 0.02 | -2.21 ± 0.35 |
|  | A264 | -0.94 ± 0.57 | -2.45 ± 0.38 | 1.43 ± 0.04 | -0.16 ± 0.02 | -2.12 ± 0.20 |
|  | S167 | -0.21 ± 0.69 | -2.31 ± 0.42 | 1.36 ± 0.27 | -0.06 ± 0.02 | -1.21 ± 0.43 |
|  | Y126 | -1.54 ± 0.22 | -0.86 ± 0.06 | 1.25 ± 0.17 | -0.07 ± 0.01 | -1.22 ± 0.11 |
|  | L234 | -1.16 ± 0.32 | -0.04 ± 0.08 | 0.19 ± 0.14 | -0.10 ± 0.03 | -1.16 ± 0.32 |
|  | F163 | -1.37 ± 0.20 | -0.87 ± 0.09 | 1.38 ± 0.21 | -0.18 ± 0.01 | -1.04 ± 0.14 |
|  | V130 | -0.75 ± 0.20 | 0.06 ± 0.03 | -0.06 ± 0.02 | -0.01 ± 0.01 | -0.77 ± 0.20 |
| *ELE*_MM_ and *VDW*_MM_ respectively represent the electrostatic and van der Waals parts of intramolecular energy under vacuum at the residue level, both of which can be predicted using molecular mechanics. *ELE*_GB_ and *VDW*_SA_ are polar and non-polar parts of solvation-free energy at the residue level, respectively. The former can be calculated by the GB algorithm, while the latter can be obtained by linear fitting with the solvent-accessible surface area. *E*_tot_ refers to the total binding energy of receptor-ligand recognition, which is obtained by summation of *ELE*_MM_, *VDW*_MM_, *ELE*_GB_ and *VDW*_SA_. | | | | | | |

**Table S3**. Activity prediction results of a series of IPD analogue inhibitors for the training and test sets from the CoMFA and CoMSIA models.

| Cmpd#^a^ | IC_50_ (nM)^b^ | pIC_50_^c^ | CoMFA | | |  | CoMSIA | | |
| --- | --- | --- | --- | --- | --- | --- | --- | --- | --- |
|  |  |  | Pred.^d^ | Res.^e^ | Error rate^f^ |  | Pred.^d^ | Res.^e^ | Error rate^f^ |
| 1 | 3.00 | 5.523 | 5.211 | -0.312 | 5.65% |  | 5.207 | -0.316 | 5.72% |
| 2 | 0.83 | 6.081 | 5.806 | -0.275 | 4.52% |  | 5.760 | -0.321 | 5.28% |
| 3 | 1.20 | 5.921 | 6.088 | 0.167 | 2.82% |  | 5.907 | -0.014 | 0.24% |
| 4 | 53.00 | 4.276 | 4.313 | 0.037 | 0.87% |  | 4.178 | -0.098 | 2.29% |
| 5 | 53.00 | 4.276 | 4.471 | 0.195 | 4.56% |  | 4.470 | 0.194 | 4.54% |
| 6 | 9.70 | 5.013 | 4.766 | -0.247 | 4.93% |  | 5.057 | 0.044 | 0.88% |
| 7 | 3.40 | 5.468 | 5.162 | -0.306 | 5.60% |  | 5.695 | 0.227 | 4.15% |
| 8 | 2.10 | 5.687 | 5.780 | 0.093 | 1.64% |  | 5.746 | 0.059 | 1.04% |
| 9 | 1.70 | 5.770 | 5.930 | 0.160 | 2.77% |  | 5.708 | -0.062 | 1.07% |
| 10 | 19.00 | 4.721 | 4.855 | 0.134 | 2.84% |  | 5.140 | 0.419 | 8.88% |
| 11 | 54.00 | 4.268 | 4.123 | -0.145 | 3.40% |  | 4.162 | -0.106 | 2.48% |
| 12 | 1.80 | 5.745 | 5.710 | -0.035 | 0.61% |  | 5.950 | 0.205 | 3.57% |
| 13 | 0.49 | 6.310 | 6.043 | -0.267 | 4.23% |  | 6.032 | -0.278 | 4.41% |
| 14 | 46.00 | 4.337 | 4.322 | -0.015 | 0.35% |  | 4.448 | 0.111 | 2.56% |
| 15 | 37.00 | 4.432 | 4.224 | -0.208 | 4.69% |  | 4.455 | 0.023 | 0.52% |
| 16 | 180.00 | 3.745 | 3.760 | 0.015 | 0.40% |  | 3.648 | -0.097 | 2.59% |
| 17 | 37.00 | 4.432 | 4.391 | -0.041 | 0.93% |  | 4.455 | 0.023 | 0.52% |
| 18 | 40.00 | 4.398 | 4.285 | -0.113 | 2.57% |  | 4.388 | -0.01 | 0.23% |
| 19 | 24.00 | 4.620 | 4.737 | 0.117 | 2.53% |  | 4.705 | 0.085 | 1.84% |
| 20 | 160.00 | 3.796 | 3.748 | -0.048 | 1.26% |  | 3.806 | 0.010 | 0.26% |
| 21 | 97.70 | 4.010 | 4.049 | 0.039 | 0.97% |  | 4.017 | 0.007 | 0.17% |
| 22 | 76.90 | 4.114 | 4.681 | 0.567 | 13.78% |  | 4.000 | -0.114 | 2.77% |
| 23 | 0.15 | 6.824 | 5.995 | -0.289 | 4.24% |  | 6.485 | -0.339 | 4.97% |
| 24 | 0.37 | 6.432 | 5.503 | -0.929 | 14.44% |  | 5.832 | -0.600 | 9.33% |
| 25 | 0.42 | 6.377 | 5.471 | -0.906 | 14.21% |  | 5.785 | -0.592 | 9.28% |
| 26 | 0.29 | 6.538 | 5.891 | -0.647 | 9.90% |  | 6.121 | -0.417 | 6.38% |
| ^a^ 1-22 and 23-26 belong to the training set and test set respectively; ^b^ IC_50_ experimental values of inhibitors ; ^c^ Negative logarithm of the experimental value IC_50_ ; ^d^ Predicted values of CoMFA and CoMSIA models ; ^e^ The absolute difference between the predicted values and experimental values in 3D-QSAR ; ^f^ Error rate of 3D-QSAR prediction. | | | | | | | | | |
